# Activity in the dorsal hippocampus-mPFC circuit modulates stress-coping strategies during inescapable stress

**DOI:** 10.1038/s12276-024-01294-z

**Published:** 2024-09-02

**Authors:** Sang Ho Yoon, Woo Seok Song, Geehoon Chung, Sang Jeong Kim, Myoung-Hwan Kim

**Affiliations:** 1 Department of Physiology and Biomedical Sciences, Seoul National University College of Medicine, Seoul, 03080 Korea; 2 Neuroscience Research Institute, Seoul National University Medical Research Center, Seoul, 03080 Korea; 3 Seoul National University Bundang Hospital, Seongnam, Gyeonggi 13620 Korea; 4 Present Address: Department of Anatomy & Neurobiology, University of California Irvine, Irvine, CA 92697 USA; 5 Present Address: Department of Physiology, College of Korean Medicine, Kyung Hee University, Seoul, 02447 Korea

**Keywords:** Cellular neuroscience, Neuronal physiology

## Abstract

Anatomical connectivity and lesion-deficit studies have shown that the dorsal and ventral hippocampi contribute to cognitive and emotional processes, respectively. However, the role of the dorsal hippocampus (dHP) in emotional or stress-related behaviors remains unclear. Here, we showed that neuronal activity in the dHP affects stress-coping behaviors in mice via excitatory projections to the medial prefrontal cortex (mPFC). The antidepressant ketamine rapidly induced c-Fos expression in both the dorsal and ventral hippocampi. The suppression of GABAergic transmission in the dHP-induced molecular changes similar to those induced by ketamine administration, including eukaryotic elongation factor 2 (eEF2) dephosphorylation, brain-derived neurotrophic factor (BDNF) elevation, and extracellular signal-regulated kinase (ERK) phosphorylation. These synaptic and molecular changes in the dHP induced a reduction in the immobility time of the mice in the tail-suspension and forced swim tests without affecting anxiety-related behavior. Conversely, pharmacological and chemogenetic potentiation of inhibitory neurotransmission in the dHP CA1 region induced passive coping behaviors during the tests. Transneuronal tracing and electrophysiology revealed monosynaptic excitatory connections between dHP CA1 neurons and mPFC neurons. Optogenetic stimulation of dHP CA1 neurons in freely behaving mice produced c-Fos induction and spike firing in the mPFC neurons. Chemogenetic activation of the dHP-recipient mPFC neurons reversed the passive coping behaviors induced by suppression of dHP CA1 neuronal activity. Collectively, these results indicate that neuronal activity in the dHP modulates stress-coping strategies to inescapable stress and contributes to the antidepressant effects of ketamine via the dHP-mPFC circuit.

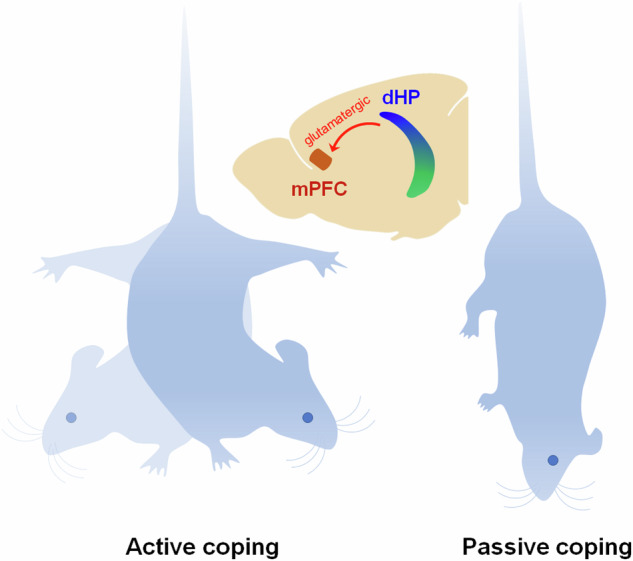

## Dorsal hippocampus neuronal activity modulates stress-coping strategies in mice

Understanding our brain’s handling of emotions and thought is vital. Recent research indicates that different sections of the hippocampus are responsible for either thought processing or emotional reactions. However, new research by Yoon et al. disputes this, demonstrating that both hippocampus sections can affect behavior via connections with the prefrontal cortex, a brain area involved in decision-making. This study, a mouse experiment, examined how a specific pathway between the dorsal hippocampus and the medial prefrontal cortex impacts behavior, especially in response to stress and depression-like symptoms. The researchers used various methods, including gene alteration and behavioral tests, to understand how changes in this pathway’s activity affect behavior. This study paves the way for future research to explore how these brain areas interact in the context of emotional and thought processing, potentially leading to more effective depression treatments.

This summary was initially drafted using artificial intelligence, then revised and fact-checked by the author.

## Introduction

Anatomical connectivity and lesion-deficit studies have shown that the hippocampus (HP) is functionally segregated along the longitudinal axis. The dorsal HP (dHP, posterior HP in primates) contributes to cognitive processing via projections to the retrosplenial cortex and septum, whereas the ventral HP (vHP, anterior HP in primates) mediates emotional processing by innervating the medial prefrontal cortex (mPFC), hypothalamus, bed nucleus of the stria terminalis (BNST), and amygdala^1–5^. In contrast to this widely accepted idea, recent neural tracing studies have shown that mPFC neurons have direct synaptic connections with the axon terminals of CA1 neurons in both the dHP and vHP^6–8^. This finding indicates that the dHP affects neuronal activity in the mPFC and modifies behaviors controlled by the mPFC. However, the behavioral role of the dHP-mPFC circuit, except for the regulation of fear memory consolidation and extinction, is still unclear^7^. In addition, the neurophysiological characteristics of the dHP-mPFC circuit are unknown.

Ketamine (an NMDAR blocker) produces rapid and sustained antidepressant effects^9^. Notably, a single ketamine administration rapidly reduced suicidal thoughts in patients with depression^10,11^ and behavioral despair in rodent models^12,13^. Although the mechanisms underlying the rapid and sustained antidepressant effects of ketamine are still unclear, increased expression and signaling of brain-derived neurotrophic factor (BDNF) are believed to trigger long-lasting changes in synaptic efficacy and neuronal morphology in the mPFC and HP^9,12–15^. Multiple hypotheses, including disinhibition-induced glutamate burst^9^, desuppression of BDNF translation by dephosphorylation of eukaryotic elongation factor 2 (eEF2)^12^, and cAMP-dependent glial BDNF release^14^, have been proposed for the mechanism responsible for ketamine-induced BDNF elevation. It has been suggested that neuronal activity in the mPFC and ventral hippocampus (vHP) is critical for the behavioral responses to ketamine^16–18^.

However, important questions remain to be addressed: Does ketamine selectively affect the ventral region of the HP? Which emotion-related behaviors are influenced by altered neuronal activity in the dHP? Which synaptic and molecular mechanisms mediate the rapid and sustained antidepressant effects of ketamine? What is the circuit mechanism by which dHP modulates this behavior?

In this study, we found that an antidepressant dose of ketamine rapidly increased the activity of neurons in the dHP in vivo. To identify the behavioral consequences of altered dHP activity, we examined the behavioral responses to altered inhibitory synaptic transmission, interneuron activity, and pyramidal neuron activity in the dHP CA1 region. Neuronal activity in the dHP affected the immobility/despair-like behavior of mice in the tail suspension and forced swim tests via excitatory projections to the mPFC. We also showed that disinhibition of CA1 neurons induced eEF2 dephosphorylation and BDNF elevation, which promoted the sustained antidepressant effect of ketamine.

## Materials and Methods

### Animals

All experiments were performed using C57BL/6N (Orient Bio, Sungnam, Korea) mice. Animals were group-housed (3−5/cage) in a specific pathogen-free facility and maintained in a climate-controlled room with free access to food and water under a 12 h light/dark cycle. Vgat-ires-Cre (Slc32a1^tm2(Cre)Lowl^ knock-in; Jackson Laboratory stock #016962) mice were backcrossed with C57BL/6N mice for at least 10 generations before use. PV-Cre knock-in (Pvalb^tm1(cre)Arbr^; Jackson Laboratory stock #017320) mice were transferred from the Institute for Basic Science (IBS, Daejeon, Korea) and backcrossed with C57BL/6N mice for two generations. CaMKIIα-Cre transgenic mice were kindly provided by Ronald Duman (Yale School of Medicine) and were originally obtained from Günter Schütz (German Cancer Research Center, Heidelberg, Germany). All animal maintenance and experiments were approved by the Institutional Animal Care and Use Committee (IACUC) of SNU.

### Behavioral analyses

All behavioral tests were performed between 10 a.m. and 6 p.m. on male mice that were at least 9 weeks old. All experimental mice were acclimated to the behavior testing room for at least 1 h prior to the beginning of testing. The testing apparatuses were cleaned with 70% ethanol between trials. Muscimol and ANA-12 were purchased from Sigma‒Aldrich (St. Louis, MO, USA). Ketamine hydrochloride was obtained from Yuhan (Seoul, Korea). Clozapine-N-oxide was acquired from Tocris (Bristol, UK).

For the tail suspension test (TST), mice were suspended by the tail from a metal hook extending from a steel bar using adhesive tape in a 3-sided chamber with opaque walls. The distance between the floor of the chamber and the steel bar was approximately 40 cm. Mice that climbed onto their tails or fell off during the test were excluded from analysis. The duration of immobility over a 6-min session was manually scored by an experienced investigator blinded to the treatment group and mouse genotype.

The forced swim test (FST) was conducted by placing mice in glass beakers (20 cm high, 15 cm in diameter) containing 25−26 °C water at a 15 cm depth. An opaque plastic divider was placed between the glass beakers. The water was changed regularly, and the beakers were cleaned between subjects. A mouse was judged to be immobile when it floated in the water without struggling or swimming and only made the minimum movements necessary to stay afloat. The total duration of immobility over the 6-min observation period was scored by an experimenter blinded to the experimental details.

The open field test (OFT) was conducted by placing the mouse in the center of an open field apparatus with opaque walls (40 × 40 × 40 cm) in a dimly lit (40 lux) room. The behavior of each mouse was monitored via video recording. The total distance traveled and time spent in the entire open field and in the center (20 × 20 cm) were calculated using video tracking software (EthoVision XT, Noldus, Netherlands).

The elevated plus maze (EPM) test was performed using a plus-shaped maze with two open and two closed arms surrounded by opaque walls (20 cm in height). The maze was elevated 50 cm above the floor, and each arm was 5 cm wide and 30 cm long. Each mouse was placed in the center of the maze, and its movement was video-recorded for 5 min.

A black acrylic circular track that was 5 cm wide, 60 cm in diameter, and elevated 50 cm above the floor was used for the elevated zero maze (EZM) test. The maze was divided into four equal quadrants, two of which were enclosed by black acrylic walls (20 cm height). Each mouse was placed in the center of one of the two closed quadrants, and its movement was video-recorded for 5 min.

The female urine sniffing test (FUST) was performed in a dimly lit room. The mice were habituated for 60 min to a sterile cotton-tipped applicator in their home cages. Each mouse was exposed to the applicator dipped in tap water for 3 min. After a 45-min interval during which the applicator was removed from the cage, the mouse was exposed for 3 min to an applicator infused with fresh urine collected from 8- to 12-week-old females. The behavior of each mouse was video-recorded, and the time spent sniffing the cotton-tipped applicator was measured.

For the novelty-suppressed feeding test, mice were food-deprived for 24 h before exposure to a novel arena (40 × 40 × 40 cm) with a small amount of food in the center. The latency to feed was measured after the video recording. Nonfeeding behaviors such as touching and sniffing were ignored. All tested mice were returned to their home cages with free access to food and water.

The TST and FST were performed under 70 lux illumination, and the other tests were conducted under 40 lux illumination.

### Constructs and virus preparation

For the Gabrg2 knockdown assay, Gabrg2 cDNA was amplified from mouse hippocampal cDNA and was cloned into a pGW1 vector. Four independent shRNA (#1: gcattggaagctcagtctactctcctgta; #2: tggaatgatggtcgagttctctacacctt; #3: ccaaggtctcctatgtcacagcaatggat; #4: tggattgaggaatacaactgaagtagtga) constructs in the pGFP-V-RS vector and a nontargeting (NT: gcactaccagagctaactcagatagtact) pGFP-V-RS plasmid were purchased from OriGene (Cat. #TL515175). For lentivirus-mediated expression, cassettes containing the human U6 (hU6) promoter-shRNA#3 and hU6-NT were amplified by PCR and then subcloned and inserted into the pLVX-DsRed-Monomer-C1 (Clontech) vector, displacing the CMV promoter-DsRed-Monomer cassette. The PGK promoter-puromycin resistance gene cassette in the vector was further replaced by the CMV-eGFP cassette from the pEGFP-C1 vector (Clontech). The sequence-verified plasmid was transfected into Lenti-X 293 T cells (TaKaRa Bio) together with the packaging plasmid psPAX2 (Addgene) and the envelope plasmid pMD2.G (Addgene) using a polyethylenimine (PEI)-based transfection protocol (total DNA to PEI µg ratio of 1:2). The pLVX-shRNA:psPAX:pMD2.G ratio was 4:1:2 (total 300 µg/245 mm square dish). Lenti-X 293 T cells were cultivated in high-glucose (4.5 g/L) Dulbecco’s modified Eagle’s medium (DMEM) supplemented with 4 mM L-glutamine, 3.7 g/L sodium bicarbonate, 10% (v/v) tetracycline-free fetal bovine serum (FBS), 1 mM sodium pyruvate, and 1% (v/v) penicillin/streptomycin. The medium was replaced with fresh medium 24 h after transfection. The lentivirus-containing medium was harvested 72 h after transfection and briefly centrifuged at 900 × g for 10 min to remove debris. The supernatant was filtered (0.45-μm pore size) for sterilization, and lentivirus particles were concentrated by 2 rounds of ultracentrifugation (10,000 × g for 2 h). The pellets were resuspended in PBS, aliquoted, and stored at −80 °C. The viral titer (10^12−13^ IU/mL) was determined by transfecting serially diluted virus stocks into HEK293T cells and analyzing the number of GFP-positive cells using a fluorescence-activated cell sorter (FACS).

AAV2/hSyn-DiO-mCherry (#50459), AAV2/hSyn-DiO-hM3Dq-mCherry (#44361), AAV2/hSyn-DiO-hM4Di-mCherry (#44362), AAV2/hSyn-DiO-EGFP (#50457), AAV5/CaMKIIa-hChR2(H134R)-mCherry (#26975), AAV5/hSyn-mCherry (#114472), and AAV9/Syn-Flex-GCaMP6f-WPRE-SV40 (#100833) were obtained from Addgene (Watertown, MA, USA). AAVDJ/hSyn1-mCherry-IRES-WGA-Cre was obtained from the Stanford University Gene Vector and Virus Core. AAV2/SST-Cre (#CV17210-AV2) was purchased from Vigene Biosciences (Rockville, MD, USA).

### Surgery and stereotaxic injection

Under deep anesthesia with a mixture of Zoletil (50 mg/kg, i.p.) and xylazine (1 mg/kg, i.p.), the mice were placed in a stereotaxic device. The skin was cut over the midline, and craniotomies were performed bilaterally over the dHP (−1.9 anteroposterior, ±1.5 mediolateral, −1.8 dorsoventral from the bregma and dura) and/or the mPFC (+1.8 anteroposterior, ±0.5 mediolateral, −2.5 dorsoventral). Either purified AAV (0.5 µL/side) or lentivirus (1 µL/side) was injected using a Hamilton syringe at a rate of 100 nL/min. After completion of the injection, the needle (33 gauge) was kept in place for an additional 10 min to allow diffusion of the injection medium and then carefully retracted to prevent backflow. The experiments were performed 3-4 weeks after the viral injections.

For the intrahippocampal infusion of muscimol, cannula assemblies consisting of a stainless-steel guide cannula (26 gauge with 2 mm) and a stylet were bilaterally implanted into the dHP (−1.9 anterior/posterior, ±1.5 medial/lateral, −1.2 dorsal/ventral) and affixed to the skull with dental cement. After surgery, the mice were allowed to recover for at least 10 days before microinjection. Intrahippocampal infusion (0.5 µL/side) was performed using a syringe pump (Pump 11, Harvard Apparatus, Holliston, MA, USA) at a rate of 100 nL/min. Stainless steel injection cannulas connected to the injection syringe by polyethylene tubing were inserted into the guide cannulas. Following microinjection, the injection cannulas were kept in place for 5 min to allow diffusion of the solution into the tissue, which was then replaced with stylets.

### Optogenetic stimulation

AAV5/CaMKIIa-hChR2(H134R)-mCherry or AAV5/hSyn-mCherry virus was injected into the dHP unilaterally, and an optic fiber (200 μm diameter) held in 1.25-mm ferrules (Thorlabs, Newton, NJ, USA) was implanted above the CA1 pyramidal layer (−1.9 anteroposterior, 1.5 mediolateral, −1.2 dorsoventral from bregma and dura). The implants were secured with metal screws and dental cement. The mice were allowed to recover for 3 weeks after surgery. A fiber-optic patch cord was connected to the brain-implanted optic fiber, and photostimulation (20 ms pulses) was delivered using a 473 nm laser diode light source (MBL-III-473 DPSS laser, CNI, Changchun, China). The light intensity (5–6 mW) was adjusted using a photodiode power sensor (S120C, Thorlabs) coupled with a power meter (PM100D, Thorlabs). dHP neurons were photostimulated (20 Hz, 1 s) every 2 min for 1 h to avoid inducing epileptic seizures. The behaviors of the mice were monitored during and after stimulation. Seizure-free mice were anesthetized and transcardially perfused with a fixative 60 min after completion of photostimulation to detect c-Fos induction in the mPFC.

### Fiber photometry

For simultaneous fiber photometry recording and optogenetic stimulation, AAV5/CaMKIIa-hChR2(H134R)-mCherry and AAV9/Syn-Flex-GCaMP6f-WPRE-SV40 were unilaterally injected into the dHP and mPFC (+1.8 anteroposterior, ±0.5 mediolateral, −2.5 dorsoventral), respectively. Optic fibers for optogenetic stimulation (200 μm diameter held in a 1.25-mm stainless steel ferrule) and fiber photometric recording (400 μm diameter, 0.39 NA, CFM14U-20, Thorlabs) were implanted above the injection site in the dHP and mPFC, respectively. The implants were secured using dental cement and a pair of metal screws anchored to the skull in the contralateral hemisphere. The mice were allowed to recover for 3 weeks after surgery.

Ratiometric fiber photometry in the mPFC was conducted using an RZ5P processor running Synapse software (Tucker-Davis Technologies, Alachua, Florida, USA), as previously described^19^. Briefly, a 405 nm LED (Doric Lenses, Quebec, Canada) was modulated at 211 Hz to detect Ca^2+^-independent isosbestic signals, which were used as reference signals to indicate motion artifacts and photobleaching. A 470 nm LED (Doric Lenses) was modulated at 531 Hz to detect Ca^2+^-dependent signals. Light from the LEDs and GCaMP6f fluorescence were passed through a Minicube instrument (FMC6, Doric Lenses) containing GFP excitation and emission filter sets. The emitted light was detected using a femtowatt photoreceiver (2151; Newport, Irvine, CA, USA). Light power (10–30 μW) was measured at the tip of the fiber-optic patch cord and adjusted using a light source device (LDFLS4, Doric Lenses) to yield the intensity of receiving light within the range of the photoreceiver. Photostimulation (10 Hz, 1 s) was triggered every 1 min using a function generator (AFG3021C, Tektronix, Beaverton, OR, USA), and the absolute time of each trigger and fluorescence signal were simultaneously recorded using the RZ5P processor. The fluorescence signals (1 kHz) were low-pass filtered with a frequency cutoff of 10 Hz and demodulated to 381 Hz using a MATLAB script. The time course of photobleaching was estimated by double exponential fitting to the fluorescence signals of the entire period^20^, and photobleaching was corrected using a custom macro written in Igor Pro (WaveMetrics, Portland, OR, USA). ΔF/F was calculated by dividing the change in the fluorescence signal by the baseline signal level.

### Electrophysiology

Parasagittal hippocampal slices (400 µm thick) or coronal mPFC slices (400 µm thick) were prepared using a vibratome (Leica, Germany) in ice-cold dissection buffer (230 mM sucrose; 25 mM NaHCO_3_; 2.5 mM KCl; 1.25 mM NaH_2_PO_4_; 10 mM D-glucose; 1.3 mM Na-ascorbate; 3 mM MgCl_2_; 0.5 mM CaCl_2_, pH 7.4 with 95% O_2_/5% CO_2_). Immediately after sectioning, the CA3 region was surgically removed from the dHP slices. The slices were allowed to recover at 36 °C for 1 h in normal artificial cerebrospinal fluid (ACSF: 125 mM NaCl; 25 mM NaHCO_3_; 2.5 mM KCl; 1.25 mM NaH_2_PO_4_; 10 mM D-glucose; 1.3 mM MgCl_2_; 2.5 mM CaCl_2_, pH 7.4, with 95% O_2_/5% CO_2_) and then maintained at room temperature.

All electrophysiological recordings were performed as described previously^21^. The slices were placed in a submerged recording chamber, which was perfused with heated (29–30 °C) ACSF. The signals were filtered at 2.8 kHz and digitized at 10 kHz using a MultiClamp 700B amplifier and Digidata 1440 A interface (Molecular Devices, San Jose, CA, USA). The data were analyzed using custom macros written in Igor Pro.

Miniature excitatory postsynaptic currents (mEPSCs) were measured at –70 mV with a pipette solution containing (in mM) 110 K-gluconate, 20 KCl, 8 NaCl, 10 HEPES, 0.5 QX-314-Cl, 4 Mg-ATP, 0.3 Na-GTP, and 10 BAPTA, adjusted to pH 7.25 and 290 mOsm/kg. Spontaneous action potentials and inhibitory postsynaptic currents (IPSCs) were blocked by tetrodotoxin (TTX, 1 µM) and picrotoxin (50 µM), respectively. For miniature IPSC (mIPSC) recording, K-gluconate in the pipette solution was replaced with equimolar KCl, and currents were measured at –70 mV in the presence of TTX (1 µM), NBQX (10 µM), and AP-5 (50 µM) in ACSF.

For measurement of the evoked EPSC/IPSC ratio, whole-cell voltage clamp recordings were made using patch pipettes (3–4 MΩ) filled with a solution containing (in mM) 130 CsMeSO4, 10 TEA-Cl, 10 HEPES, 4 Mg-ATP, 0.3 Na-GTP, 5 QX-314-Cl, and 10 EGTA, adjusted to pH 7.25 and 290 mOsm/kg. The synaptic responses were evoked at 0.05 Hz with an ACSF-filled broken glass pipette (0.3–0.5 MΩ) placed in the proximal region of the stratum radiatum. The mean AMPAR-mediated EPSCs were obtained by averaging 30–40 traces recorded at –57 mV. The stimulation intensity was adjusted to yield a 100–300 pA EPSC peak amplitude. For isolation of the GABA_A_R-mediated currents, 30–40 traces of synaptic currents were recorded at +3 mV. The series resistance and seal resistance were monitored, and data were discarded if they changed by more than 20% during the recordings.

The membrane potentials of dHP interneurons were recorded under whole-cell current clamp mode with a pipette solution containing (in mM) 110 K-gluconate, 20 KCl, 8 NaCl, 10 HEPES, 4 Mg-ATP, 0.3 Na-GTP, and 0.5 EGTA, adjusted to pH 7.25 and 290 mOsm/kg. Neurons displaying an unstable resting potential at the beginning or during recording were discarded.

Light-evoked excitatory postsynaptic potentials (EPSPs), inhibitory postsynaptic potentials (IPSPs), and action potentials in the mPFC cells were measured using a pipette solution containing (in mM) 130 K-gluconate, 8 NaCl, 10 HEPES, 4 Mg-ATP, 0.3 Na-GTP, and 0.5 EGTA, adjusted to pH 7.25 and 290 mOsm/kg. The experiments were conducted at least four weeks after virus injection to allow terminal expression. The ipsilateral mPFC was identified by mCherry fluorescence signals, and an optic fiber with a blue laser was directly positioned above the slices for photostimulation. Light-evoked EPSCs, IPSCs, and NMDAR-mediated currents were measured using the same pipette solution that was used to measure the evoked EPSC/IPSC ratio in CA1 pyramidal neurons. NMDAR-mediated currents were measured at a holding potential of –40 mV in the presence of picrotoxin (50 µM) and NBQX (10 µM) in the ACSF.

All reagents were purchased from Sigma‒Aldrich (St. Louis, MO, USA), except QX-314-Cl, CNO, NBQX, and AP-5, which were purchased from Tocris (Bristol, UK).

### Immunohistochemistry and western blotting

Immunohistochemistry and western blotting analyses were performed as described previously^21^. Briefly, mice were deeply anesthetized with diethyl ether and transcardially perfused with heparinized (10 U/mL) phosphate-buffered saline (PBS), followed by PBS-buffered 4% (w/v) paraformaldehyde (PFA). The brains were removed, postfixed in 4% PFA for 48 h at 4 °C, and cut into 100 μm coronal sections using a vibratome (VT1200S, Leica, Germany). The sections were postfixed (1 h), permeabilized with 0.3% (v/v) Triton X-100 in PBS, and incubated in blocking buffer (5% normal goat serum, 5% horse serum, 5% donkey serum, and 0.5% BSA in PBS) for 2 h. The sections were successively incubated with primary anti-PV (Swant, Cat. #PV27 and Millipore Cat. #MAB1572), anti-SST (Santa Cruz Biotechnology, Cat. #sc-55565), anti-GAD67 (Millipore, Cat. #MAB5406), anti-mCherry (Abcam, Cat. #ab167453 and Thermo Fisher, Cat. #M11217), anti-GFP (Synaptic Systems, Cat. #132 004), anti-c-Fos (Cell Signaling Technology, Cat. #2250S), and anti-NeuN antibodies (Millipore, Cat. #ABN78 and Cat. #MAB377) overnight at 4 °C and fluorescence (Cy3, Alexa Fluor 647 or FITC: Jackson ImmunoResearch Laboratories, PA, USA)-conjugated secondary (3 h at room temperature) antibodies. Between each step, the sections were rinsed 3 times for 10 min with PBS. Images were acquired using an FV3000 confocal laser scanning microscope and processed with the FV31S-SW Viewer (Olympus, Japan). Wide-field images of entire brain sections were acquired using a TCS SP8 confocal microscope and Leica Application Suite X (Leica, Germany).

For western blotting, mouse hippocampi were homogenized in buffer (320 mM sucrose, 10 mM Tris-HCl, 5 mM EDTA, pH 7.4) containing phosphatase inhibitor cocktail (GenDEPOT, TX, USA, Cat. #P3200) and proteinase inhibitor cocktail (Sigma‒Aldrich, MO, USA, Cat. #P8340). Proteins were separated by SDS‒PAGE and transferred to nitrocellulose membranes. The membranes were subsequently incubated with primary and horseradish peroxidase (HRP)-conjugated secondary antibodies (Jackson ImmunoResearch Laboratories, PA, USA). Signals were detected by enhanced chemiluminescence (GE Healthcare, UK) and quantified using MetaMorph software (Molecular Devices, CA, USA). The following primary antibodies were obtained from commercial suppliers: anti-ERK (Cat. #9102S), anti-p-ERK (Cat. #9106S), anti-eEF2 (Cat. #2332S), anti-p-eEF2 (Cat. #2331S), anti-mTOR (Cat. #2972S), and anti-p-mTOR (Cat. #2971S) from Cell Signaling Technology; BDNF (Cat. #ab108319) from Abcam; Gabrg2 (Cat. #224 003) from Synaptic Systems; tGFP (Cat. #TA150041) from Origene; and α-tubulin (Cat. #T5168) from Sigma‒Aldrich.

### Quantification and statistical analysis

Statistical analyses were performed using Igor Pro (WaveMetrics) and SPSS (IBM, Armonk, USA). The normality of the collected data was determined using the Shapiro‒Wilk test. The Mann‒Whitney test was used to compare non-normally distributed samples. Samples that satisfied a normal distribution were compared using two-tailed Student’s *t* tests. For multiple groups, one-way ANOVA followed by Tukey’s honestly significant difference (HSD) post hoc test was used to compare the samples. All bar graphs in the figures show the mean ± standard error of the mean (SEM). The levels of significance are indicated as follows: *P < 0.05, **P < 0.01, ***P < 0.001, n.s., not significant (*p* ≥ 0.05). The number of cells, slices, or animals used for each experiment and the statistical analyses are provided in Supplementary Table 1.

## Results

### An antidepressant dose of ketamine rapidly increases c-Fos expression in both the dHP and vHP

**Fig. 1:**
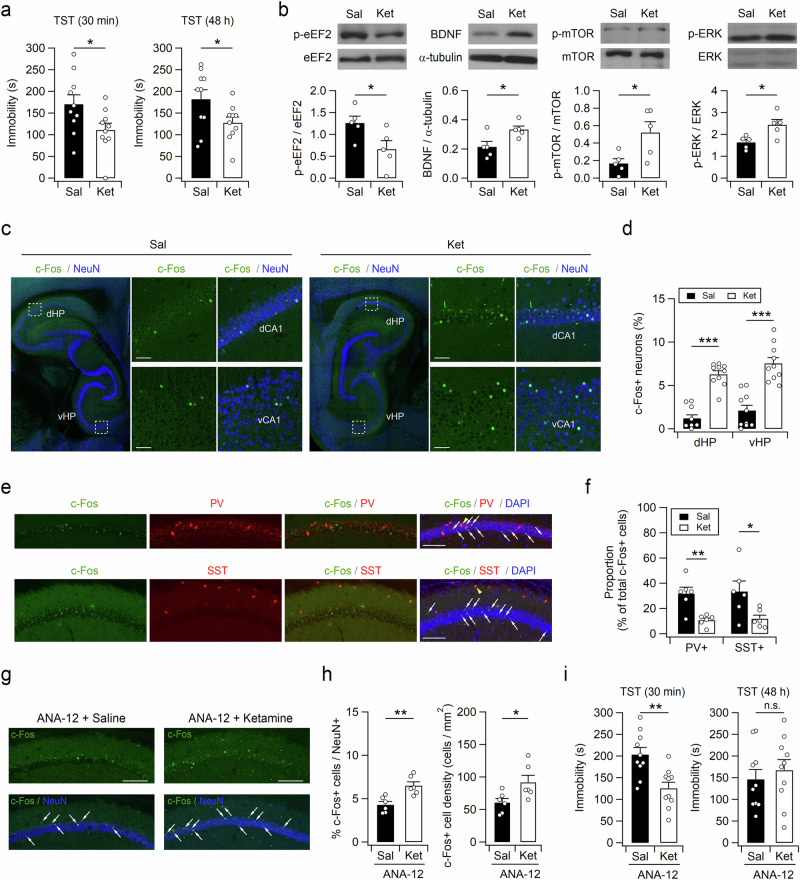
Ketamine induces rapid c-Fos induction in the dHP and vHP. **a** A single ketamine injection induces rapid and long-lasting antidepressant effects. A reduction in immobility in the TST was observed 30 min (left) and 48 h (right) after ketamine injection. The tests were performed on the same cohorts of animals. **b** Representative western blots (top) and quantification (bottom) of proteins in hippocampal lysates obtained 30 min after ketamine injection. Ketamine rapidly activates the eEF2, BDNF, mTOR, and ERK signaling pathways in the hippocampus. Uncropped blots are presented in Supplementary Fig. 12. **c** Immunohistochemical staining of sagittal hippocampal sections with an anti-c-Fos antibody showing rapid activation of neurons in the dHP and vHP by ketamine. For avoidance of possible effects of the novel context on hippocampal neurons, the mice were administered saline (Sal) or ketamine (Ket) and anesthetics (1 h after Sal or Ket injection) in their home cages in the vivarium. (Insets) Expanded images of regions corresponding to the dotted box in the left panel. dCA1, dorsal CA1; vCA1, ventral CA1. **d** Quantification of cells expressing c-Fos in the dHP and vHP CA1 regions. **e** dHP sections from ketamine-treated mice were costained with antibodies against c-Fos/PV (top) or c-Fos/SST (bottom). Yellow arrowheads indicate c-Fos+ /PV+ (top) or c-Fos+ /SST+ (bottom) cells. The white arrows indicate c-Fos+ /PV− (top) or c-Fos+/SST− (bottom) cells. **f** The number of cells expressing c-Fos/PV or c-Fos/SST was measured as the percentage of total c-Fos-positive cells in the dHP CA1 region in the saline- or ketamine-treated mice. **g** Ketamine increased the expression of c-Fos in the dHP CA1 region in the mice intraperitoneally administered ANA-12 (30 min before Sal or Ket injection). The white arrows indicate c-Fos+/NeuN+ cells. **h** Quantification of c-Fos+ cells in the dHP CA1 region in the ANA-12-pretreated mice receiving saline or ketamine. **i** Pretreatment (30 min before ketamine injection) with ANA-12 blocks the sustained but not acute antidepressant effects of ketamine. Bar graphs represent the time spent immobile during the 6-min TST performed 30 min (left) and 48 h (right) after ketamine injection. The tests were performed on the same cohorts of animals. (**a**, **b**, **d**, **f**, **h**, and **i**) *p < 0.05, **p < 0.01, ***p < 0.001, n.s., not significant (*p* ≥ 0.05). Scale bars, 50 (**c**) and 100 µm (**e** and **g**).

The rapid-acting antidepressant ketamine (1 µM) disinhibits CA1 pyramidal neurons and subsequently increases their activity in hippocampal slices^22^. However, it remains unclear whether an antidepressant dose of ketamine increases hippocampal neuronal activity in vivo. First, we determined the antidepressant dose of ketamine using the tail suspension test (TST). Intraperitoneal (i.p.) administration of 5 mg/kg ketamine produced rapid and long-lasting antidepressant effects in mice (Fig. 1a). The decreased immobility observed during the TST 30 min after ketamine administration was unlikely to have arisen from hyperlocomotion or the dissociative effects of ketamine^13^, as the increased locomotor activity did not last for more than 10 min in the open field test (OFT; Supplementary Fig. 1a and 1b).

We then determined the effects of ketamine on neuronal activity in the hippocampus. Ketamine administration (5 mg/kg, i.p.) induced rapid eEF2 dephosphorylation (Fig. 1b), indicating increased neuronal activity^23^. In addition, increased expression of the mature isoform of BDNF and activation of its downstream signaling pathways, including phosphorylation of mammalian target of rapamycin (mTOR) and ERK, were observed in the hippocampi of the ketamine-treated mice. The ketamine-induced increase in the activity of hippocampal neurons was further confirmed by c-Fos immunohistochemical staining. The number of cells expressing c-Fos, an indicator of neuronal activation, significantly increased in both the hippocampus and mPFC within 1 h of ketamine injection (Fig. 1c, d, and Supplementary Fig. 1c-e). Interestingly, in contrast to previous studies that reported acute effects of ketamine in the vHP^18,24^, an increase in the number of c-Fos+ cells was observed in both the dorsal and ventral hippocampi of the ketamine-treated mice. Moreover, most c-Fos+ cells in the CA1 area were distributed in the principal cell layer and did not exhibit immunoreactivity for parvalbumin (PV) or somatostatin (SST) (Fig. 1e and f). We observed no significant difference in the number (mean ± SEM) of cells expressing either c-Fos/PV (15.25 ± 5.43 vs. 9.87 ± 1.81 cells/mm^2^; t_(10)_ = 1.89 and *p* = 0.087 according to Student’s t test) or c-Fos/SST (11.34 ± 2.08 vs. 6.91 ± 1.24 cells/mm^2^; t_(10)_ = 1.82 and *p* = 0.097 according to Student’s t test) between the saline- and ketamine-treated mice, whereas the percentage of PV or SST neurons among all c-Fos+ cells was significantly decreased by ketamine (Fig. 1f).

We wondered whether the rapid ketamine-induced increase in c-Fos expression in hippocampal principal cells is associated with the activation of BDNF signaling. Mature BDNF exhibits a greater affinity for the TrkB receptor than for the p75 neurotrophin receptor, and the cellular effects of mature BDNF are mediated mainly via the TrkB receptor^25,26^. Because the TrkB receptor antagonist ANA-12 penetrates the blood‒brain barrier and reaches its peak concentration in the mouse brain as early as 30 min after i.p. injection^27^, we pretreated mice with ANA-12 (0.5 mg/kg, i.p.) 30 min before ketamine injection. Ketamine rapidly increased hippocampal neuronal activity in the ANA-12-pretreated mice, as revealed by the increased c-Fos expression in CA1 pyramidal neurons (Fig. 1g, h). Consistent with this observation, a rapid antidepressant effect was found in the ANA-12-pretreated mice 30 min after ketamine treatment (Fig. 1i). This finding is consistent with previous results showing that pretreatment (30 min) with intracerebroventricular administration of K252a, another TrkB receptor antagonist, did not block the antidepressant effect in the TST at 30 min after ketamine treatment^28^. Intriguingly, the antidepressant effects of ketamine did not persist for 48 h in the ANA-12-pretreated mice (Fig. 1i), indicating that elevated BDNF levels and the activation of downstream signaling pathways are responsible for the long-lasting, but not acute, antidepressant effects of ketamine^12,29,30^.

### Altered synaptic inhibition in the dHP affects immobility during the TST and FST

**Fig. 2:**
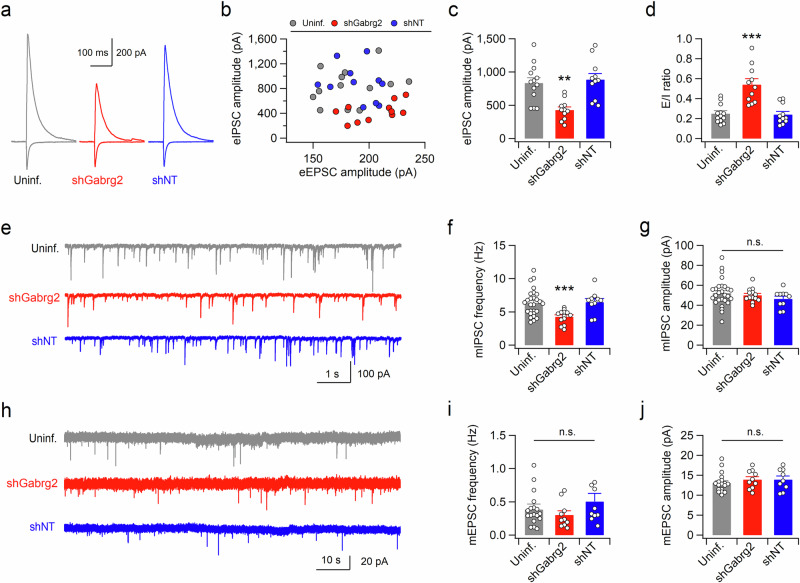
Knockdown of Gabrg2 reduces synaptic inhibition in dHP CA1 pyramidal neurons. **a** Representative traces of evoked IPSCs (upward deflections at +3 mV) and EPSCs (downward deflections at −57 mV) recorded from dHP CA1 pyramidal neurons infected with virus expressing Gabrg2-shRNA#3 (shGabrg2) or shNT and nearby uninfected (uninf.) cells. **b** The peak amplitudes of eIPSCs are plotted against the peak eEPSC amplitudes. Summary of peak eIPSC amplitudes (**c**) and the excitation-inhibition (E/I) ratios (**d**) in dHP CA1 pyramidal neurons. **e** Sample traces of mIPSCs recorded in CA1 pyramidal neurons. **f** Gabrg2 knockdown reduced the frequency of mIPSCs. **g** Summary of the mean amplitudes of mIPSCs obtained from uninfected and virus-infected (shGabrg2 or shNT) CA1 neurons. **h**−**j** Gabrg2 knockdown did not affect mEPSCs. Sample traces (**h**), mean frequencies (**i**), and mean amplitudes (**j**) of mEPSCs recorded in CA1 pyramidal neurons infected with virus expressing shGabrg2 or shNT and nearby uninfected neurons. (**c**, **d**, **f**, **g**, **i**, and **j**) **p < 0.01, ***p < 0.001, n.s., not significant (*p* ≥ 0.05).

Because ketamine administration rapidly increased neuronal activity in both the dHP and vHP, we investigated whether neuronal activity in the dHP affected depression- or emotion-related behavior in mice. Synaptic γ-aminobutyric acid A receptors (GABA_A_Rs) contain one of three γ subunits (γ_1_ – γ_3_), and the γ_2_ subunit encoded by the *Gabrg2* gene plays a critical role in clustering major postsynaptic GABA_A_Rs and is widely expressed in both excitatory and inhibitory neurons in the hippocampus^31,32^. To mimic the increased neuronal activity caused by ketamine-induced disinhibition of CA1 neurons, we virally infected CA1 neurons in the dHP with a short hairpin RNA (shRNA) sequence targeting *Gabrg2* that had the highest target knockdown activity (Gabrg2-shRNA#3, shGabrg2) among the four different knockdown vectors (Supplementary Fig. 2). Next, we recorded evoked EPSCs at the reversal potential of IPSCs using a low-chloride (15 mM) pipette solution and then recorded IPSCs at the reversal potential of EPSCs in the same cell (Supplementary Fig. 3). Compared with those infected with nontargeting shRNA (shNT) or not infected, CA1 pyramidal neurons infected with shGabrg2 exhibited reduced amplitudes of evoked IPSCs and increased EPSC/IPSC ratios (Fig. 2a–d). Consistent with the reduced synaptic inhibition, Gabrg2 knockdown significantly decreased the frequency, but not the amplitude, of mIPSCs in CA1 neurons (Fig. 2e–g). In contrast, neither the frequency nor the amplitude of mEPSCs was affected by Gabrg2 knockdown (Fig. 2h–j). These results rule out nonspecific neuronal defects induced by shRNA expression or lentivirus infection and exclude homeostatic downregulation of glutamatergic neurotransmission, which is observed in *Gabrg2* mutant mice^33^. Normal mIPSCs and mEPSCs in CA1 neurons expressing shNT further supported the specificity of Gabrg2 knockdown.

**Fig. 3:**
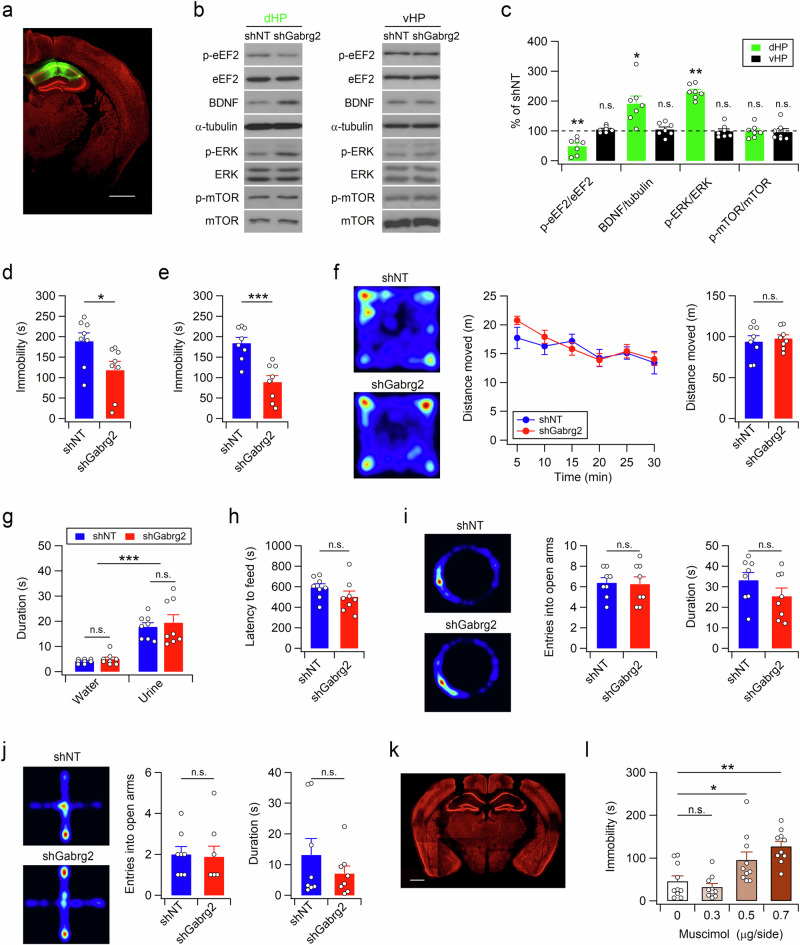
Suppression and potentiation of synaptic inhibition in the dHP induce opposite effects on despair-like behavior in mice. **a** Coronal brain section from a mouse expressing shGabrg2 in the dHP CA1 area. Sections were costained for EGFP (green) and the neuronal marker NeuN (red). Scale bar, 1 mm. Representative western blots (**b**) and quantification (**c**) of protein levels in the dHP and vHP. shNT or shGabrg2 was expressed in the mouse dHP CA1 region. Uncropped blots are presented in Supplementary Fig. 12. shRNA-mediated suppression of Gabrg2 expression in dHP CA1 neurons reduces the immobility time of mice in both the TST (**d**) and FST (**e**). **f** Representative images showing the exploration path during the entire period (left) and quantification of distance moved (middle, right) in the OFT. **g** Mean sniffing duration of water and female urine in the female urine sniffing test. **h** Knockdown of Gabrg2 in the dHP did not affect the latency to feed in a novel environment. Activity path (left), number of entries into the open arms (middle), and time spent in the open arms (right) during the elevated zero maze test (**i**) and elevated plus maze test (**j**). **k** Representative image showing cannula placement in the mouse brain after muscimol infusion. The sections were immunostained with anti-NeuN antibodies. Scale bar, 1 mm. **l** Hippocampal infusion of muscimol increased TST immobility time in a dose-dependent manner. (**c**−**j,** and **l**) *p < 0.05, **p < 0.01, ***p < 0.001, n.s., not significant (*p* ≥ 0.05).

Unexpectedly, the dHP-specific suppression of inhibitory synaptic transmission induced molecular changes similar to those induced by ketamine administration, except for mTOR, in the dHP, leaving the vHP intact (Fig. 3a–c and Supplementary Fig. 4). These observations suggest that disinhibition is sufficient to activate eEF2 and elevate BDNF levels in the dHP.

Next, we examined the behavioral effects of the increased neuronal activity in the dHP. The mice infected with the shGabrg2-expressing virus in the dHP spent significantly less time immobile in both the TST and forced swim test (FST) than did those infected with the shNT virus (Fig. 3d, e). These results suggested that the increased dHP neuronal activity caused by disinhibition was sufficient to induce active coping behaviors in response to inescapable stress. However, the mice expressing the shGabrg2 vector in the dHP behaved normally in the open field test, female urine sniffing test, novelty-suppressed feeding test, elevated zero maze test, and elevated plus maze test (Fig. 3f–j). These observations indicate that neuronal activity in the dHP mainly affects despair/helplessness rather than anxiety-related or reward-seeking behavior. We wondered whether the potentiation and suppression of synaptic inhibition in dHP CA1 neurons had opposite effects on TST immobility time in mice. Bilateral infusion of muscimol, a GABA_A_R agonist, into the dHP CA1 region of mice increased immobility time in a dose-dependent manner during the TST (Fig. 3k, l), further confirming that neural activity in the dHP regulates stress-coping behaviors.

### Pharmacogenetic manipulation of CA1 interneuron activity in the dHP modulates stress-coping behaviors

**Fig. 4:**
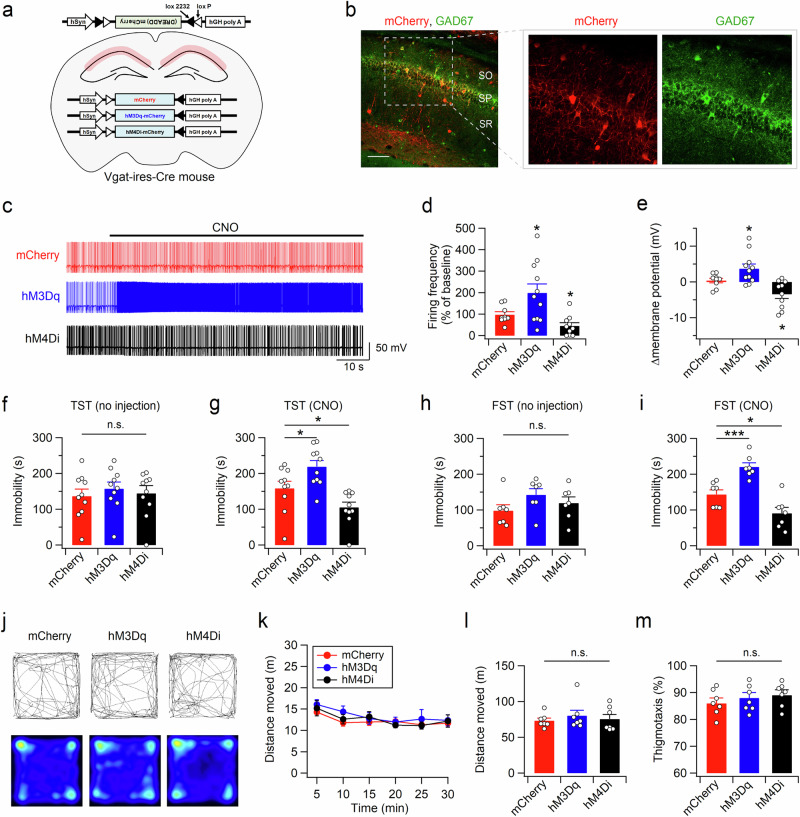
Activity of CA1 interneurons in the dHP affects TST and FST behaviors. **a** Cre-dependent expression of mCherry, hM3Dq-mCherry, or hM4Di-mCherry in dHP CA1 interneurons. **b** Double immunostaining of hippocampal sections with mCherry (red) and GAD67 (green) antibodies revealed interneuron-specific expression of mCherry in the CA1 subfield of Vgat-Cre mice. Scale bar, 100 µm. SO, stratum oriens; SP, stratum pyramidale; SR, stratum radiatum. **c** Representative voltage traces recorded from interneurons expressing mCherry, hM3Dq-mCherry, or hM4Di-mCherry before and during the bath application of CNO (5 µM). **d** Quantification of the effects of CNO on the firing rate. The firing rates under CNO are expressed as a percentage of the baseline (absence of CNO) values. **e** Changes in the membrane potential in response to CNO application are summarized. **f**–**i** Bar graphs representing the total time spent immobile during the TST (**f**, **g**) and FST (**h**, **i**). In the absence of CNO, DREADD expression did not affect despair-like behavior in the TST (**f**) or FST (**h**). CNO-induced activation or suppression of CA1 interneurons expressing DREADDs bidirectionally modified immobility time in the TST (**g**) and FST (**i**). CNO was administered 30 min and 1 h before the TST and FST, respectively. **j** Sample path recordings during the first 5 min (top) and entire 30-min period (bottom) in the OFT. **k** The open-field activities of the control (mCherry) and DREADD-expressing mice at 5-min intervals were measured 30 min after CNO injection. Quantification of the total distance moved (**l**) and thigmotaxis (**m**) during the OFT. **d**−**i**, **l**, and **m** *p < 0.05, ***p < 0.001, n.s., not significant (*p* ≥ 0.05).

Gabrg2 knockdown induced not only synaptic disinhibition but also molecular changes in the dHP. To selectively manipulate synaptic inhibition during behavioral testing, we expressed excitatory (hM3Dq) or inhibitory (hM4Di) designer receptors exclusively activated by designer drugs (DREADDs) in the dorsal CA1 region of the Vgat-ires-Cre knock-in (Vgat-Cre) mice using an adeno-associated virus (AAV) carrying Cre-dependent (double-floxed inverse orientation, DiO) DREADD receptor expression vectors (Fig. 4a). mCherry signals were restricted to GAD67-positive inhibitory neurons (Fig. 4b), including PV- and SST-expressing neurons, in the dHP (Supplementary Fig. 5). Using slice electrophysiology, we confirmed that clozapine-N-oxide (CNO) can control the activity of DREADD-expressing neurons (Fig. 4c–e).

The expression of DREADDs in inhibitory neurons in the dHP did not affect behaviors in the TST or FST. In the absence of CNO, the mice in all groups spent a similar amount of time immobile in both the TST (Fig. 4f) and FST (Fig. 4h). Interestingly, CNO injection substantially increased the immobility time of the hM3Dq-expressing mice in the TST (Fig. 4g) and FST (Fig. 4i). In contrast, the hM4Di-expressing mice exhibited reduced immobility time in the TST and FST following CNO injection. However, CNO injection did not significantly change the immobility time of the mCherry-expressing mice (TST: t_(9)_ = − 0.726, *p* = 0.487; FST: t_(6)_ = − 2.418, *p* = 0.052; paired *t* test). The CNO-induced behavioral changes in the TST and FST were unlikely to arise from altered general activity or anxiety, as the hM3Dq- and hM4Di-expressing mice showed locomotor activity and thigmotaxis levels similar to those of the mCherry-expressing mice in the OFT (Fig. 4j–m).

**Fig. 5:**
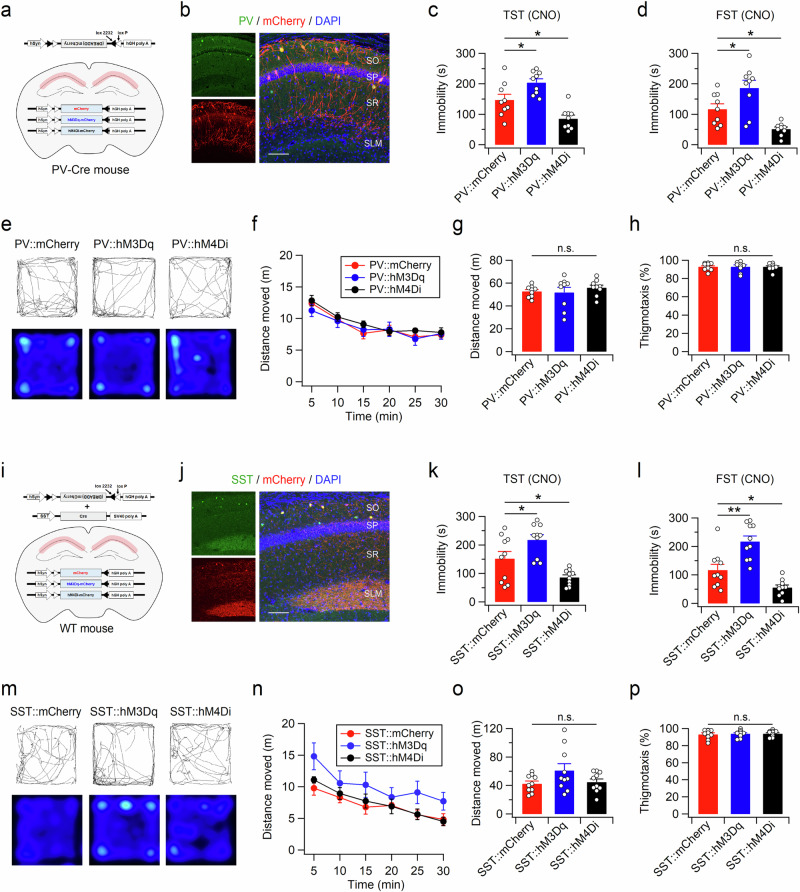
Pharmacogenetic manipulation of PV or SST interneurons in the dHP CA1 area is sufficient to modify stress-coping behavior. **a** Cre-dependent expression of mCherry, hM3Dq-mCherry, or hM4Di-mCherry in PV-positive interneurons. **b** Immunohistochemical staining of hippocampal sections from PV-Cre mice showing the expression of mCherry in PV-expressing cells in the dHP CA1 region. The immobility times in the TST (**c**) and FST (**d**) of PV-Cre mice infected with DiO-mCherry, DiO-hM3Dq-mCherry or DiO-hM4Di-mCherry. The TST and FST were measured 1 h and 30 min, respectively, after CNO injection. The TST was performed immediately after completion of the OFT (**e**–**h**). The intertest interval between the TST and FST was 7 days. **e** Example path recordings of PV-Cre mice during the first 5 min (top) and entire 30 min (bottom) of the OFT. CNO was administered 30 min before the test. Quantification of the distance moved across 5-min time bins (**f**), the entire 30-min period (**g**), and thigmotaxis (**h**) in the open field box. **i** Schematic depiction of Cre-dependent expression of mCherry, hM3Dq-mCherry, or hM4Di-mCherry in SST interneurons. **j** Expression pattern of mCherry in the hippocampal CA1 region of the WT mice coinfected with AAV-DiO-mCherry and AAV-SST-Cre. **k**–**p** Same as **c**–**h** but for mice expressing mCherry or DREADD-mCherry in SST neurons in the dHP CA1 region. (**c**, **d**, **g**, **h**, **k**, **l**, **o**, and **p**) *p < 0.05, **p < 0.01, n.s., not significant (*p* ≥ 0.05). Scale bars, 100 µm (**b**, **j**).

We further examined whether a specific type of hippocampal interneuron is associated with stress-coping behaviors. After the injection of AAV2-hSyn-DiO-DREADDs-mCherry into the dHP CA1 region of the PV-Cre knock-in (PV-Cre) mice, mCherry signals were mainly observed in PV-positive cells (Fig. 5a, b, and Supplementary Fig. 6a–c). A small proportion of mCherry-expressing cells were immunostained with anti-SST antibodies, indicating DREADD expression in cells expressing both PV and SST (Supplementary Fig. 6b). Wild-type (WT) mice infected with a mixture of AAV-SST-Cre and AAV2-hSyn-DiO-DREADDs-mCherry predominantly expressed DREADD-mCherry in SST-expressing cells (Fig. 5i, j, and Supplementary Fig. 6d–f). However, pharmacogenetic manipulation of both types of interneurons produced similar effects on immobility time in the TST (Fig. 5c, k) and FST (Fig. 5d, l). Activation of PV- or SST-expressing cells increased immobility time, whereas suppression of either type of interneuron decreased despair-like behaviors. Consistent with the pharmacogenetic manipulation of Vgat-positive interneurons, altered activity in PV- or SST-positive cells did not significantly affect the behavior of the mice in the OFT (Fig. 5e–h and m–p). Collectively, these results indicate that changes in CA1 interneuron activity are sufficient to modulate stress-coping behavior.

### Glutamatergic fibers from dHP CA1 neurons increase neuronal activity in the mPFC

**Fig. 6:**
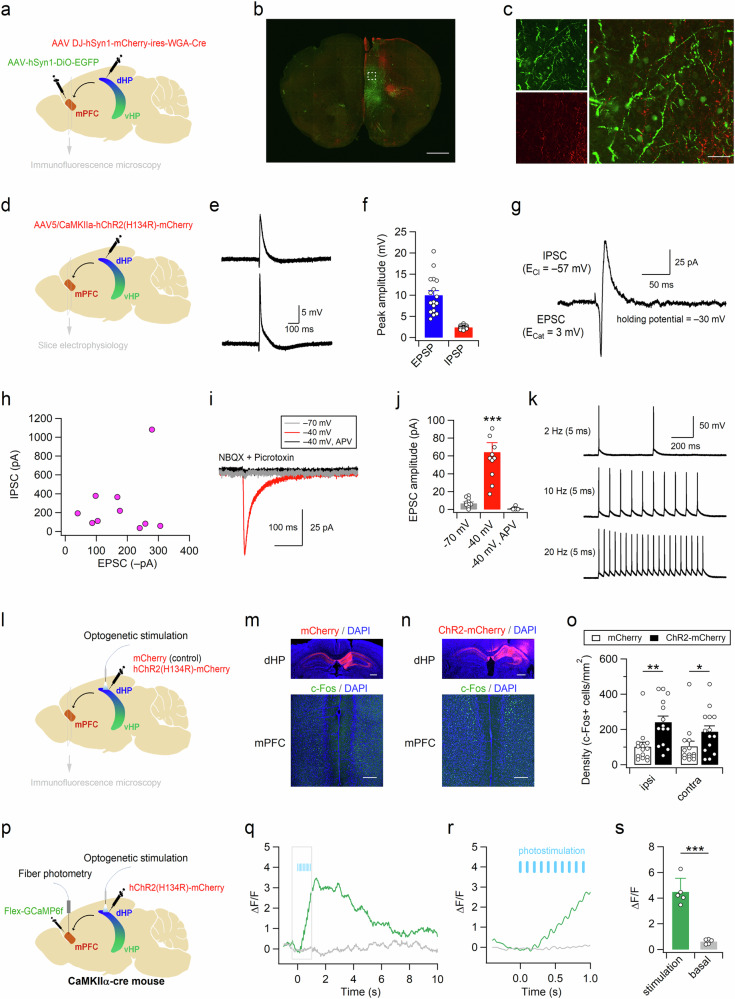
Activity of mPFC neurons is increased through monosynaptic glutamatergic projections from the dHP CA1 region. **a** Experimental design for transneuronal labeling of the dHP-mPFC circuit. AAVs carrying mCherry-ires-WGA-Cre and DiO-EGFP were unilaterally injected into the dHP and mPFC, respectively. **b** Transneuronal labeling of the dHP-mPFC circuit reveals dHP projections (red, mCherry) and dHP-recipient neurons (green, EGFP) in the mPFC. **c** Higher-resolution view of the rectangular area (**b**). **d** Experimental design for optogenetic stimulation of dHP axons and electrophysiological recording in mPFC slices. Sample traces (**e**) and mean amplitudes (**f**) of postsynaptic potentials in mPFC neurons. Optogenetic stimulation of dHP axons induces depolarization and subsequent hyperpolarization in mPFC neurons. Sample trace (**g**) showing two (inward and subsequent outward) types of postsynaptic currents (PSCs) and peak amplitudes of PSCs (**h**) in mPFC neurons. The reversal potentials of the EPSC and IPSC were 3 mV and −57 mV, respectively, and the holding potential was −30 mV. **i**, **j** Light-evoked PSCs were detected at −40 mV and blocked by AP-5. NBQX and picrotoxin were present in the bathing solution. **j** The peak amplitudes of PSCs measured under each condition are summarized. **k** Sample traces showing AP firing in mPFC neurons induced by strong stimulation of dHP axons. **l** ChR2-mCherry or mCherry was unilaterally expressed in the dHP CA1 area. Mice received 30 trains (20 Hz, 1 s) of light stimulation (20 ms, 5-6 mW) with 2-min intertrain intervals. Brain sections were collected from mice expressing mCherry (**m**) or ChR2-mCherry (**n**), and mCherry signals in the dHP (top) and c-Fos+ cells in the mPFC (bottom) were visualized by immunohistochemical staining. **o** Summary of the c-Fos+ cell density in the mPFC. **p** ChR2 and GCaMP6f were expressed in dHP CA1 neurons and mPFC CaMKII-expressing cells, respectively. Cannulas for optical stimulation and recording were implanted into the dHP and mPFC, respectively. **q** Representative fluorescence ΔF/F traces recorded in the mPFC during the peristimulation (green) and the baseline activity (gray, midpoint between each stimulation). Light stimulation (5 mW, 10 Hz, 1 s) is indicated by blue bars, and traces are averaged from 16 trials shown in Supplementary Fig. 9c. **r** Expanded scale of the rectangular area (**q**). **s** Peak amplitudes of averaged (10-20 trials) ΔF/F traces collected from 5 different animals are summarized. (**j**, **o**, and **s**) *p < 0.05, **p < 0.01, ***p < 0.001, n.s., not significant (*p* ≥ 0.05). Scale bars, 1 mm (**b**), 50 μm (**c**), and 500 μm (**m**, **n**).

We investigated whether altered activity in dHP CA1 pyramidal neurons modifies despair-like behaviors through synaptic connections to the mPFC. To elucidate the anatomic and neurophysiological characteristics of the dHP-mPFC circuit, we performed wheat germ agglutinin (WGA) transneuronal tracing by unilaterally injecting AAV-hSyn1-mCherry-IRES-WGA-Cre and AAV-hSyn-DiO-EGFP into the dHP CA1 and mPFC regions, respectively (Fig. 6a). As the genetically encoded transneuronal tracer WGA is transferred preferentially in the anterograde direction^34,35^, WGA and Cre recombinase fusion protein (WGA-Cre) are transported to the postsynaptic cells of dHP CA1 neurons, leaving mCherry in the infected cells. Indeed, mCherry-labeled axonal projections of dHP CA1 neurons were found in the mPFC and septal nuclei (Fig. 6b, c and Supplementary Fig. 7). In addition, EGFP signals were observed in the somata, dendrites, and axons of neurons in the mPFC (Fig. 6b, c and Supplementary Fig. 8). We then expressed hChR2 in dHP excitatory neurons and examined the neurophysiological characteristics of the dHP-mPFC circuit (Fig. 6d). Optogenetic stimulation of dHP axons induced depolarization and subsequent hyperpolarization of mPFC neurons (Fig. 6e, f). When we measured dual PSCs at a holding potential of −30 mV, inward EPSCs were followed by outward IPSCs (Fig. 6g, h), suggesting that IPSCs were induced by disynaptic inhibition of neighboring neurons in the mPFC. EPSCs induced by optogenetic stimulation of dHP axons were monosynaptic glutamatergic currents because NMDAR-mediated EPSCs were detected by depolarizing mPFC neurons to −40 mV in the presence of NBQX and picrotoxin in the bath solution (Fig. 6i, j). Consistent with these observations, increasing the duration or intensity of illumination generated action potentials in the mPFC neurons (Fig. 6k). These results indicate that the mPFC-projecting CA1 neurons in the dHP are glutamatergic.

We investigated the effect of dHP stimulation on mPFC neuronal activity in vivo. Interestingly, unilateral optogenetic stimulation of dHP CA1 pyramidal neurons increased c-Fos expression in both the ipsilateral and contralateral mPFC neurons (Fig. 6l–o). Increased activity in the contralateral hippocampus through interhippocampal connections may have increased the c-Fos expression in the contralateral mPFC. To determine the effects of dHP synaptic inputs on the activity of mPFC excitatory neurons in freely behaving mice, we monitored calcium signals in CaMKII-expressing neurons in the mPFC using fiber photometry. Fluorescence signals in these neurons increased rapidly in response to optogenetic stimulation of dHP CA1 pyramidal neurons and returned to basal levels within a few seconds (Fig. 6p–s and Supplementary Fig. 9). Collectively, these ex vivo and in vivo results suggested that dHP CA1 neurons increase mPFC neuronal activity via glutamatergic and monosynaptic connections.

### The dHP-mPFC circuit regulates coping strategies during inescapable stress

**Fig. 7:**
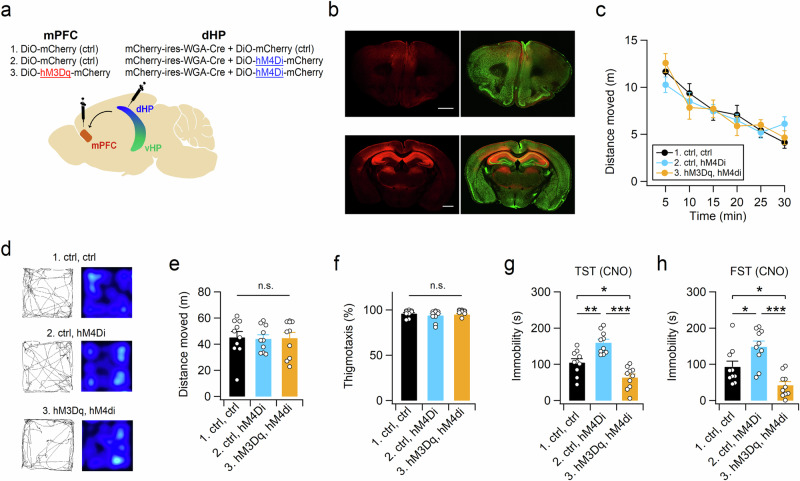
Activity manipulation of dHP-recipient mPFC neurons modifies TST and FST behaviors. **a** Schematic of chemogenetic suppression of dHP CA1 neurons with or without chemogenetic activation of dHP-recipient mPFC neurons. AAVs were bilaterally expressed in the dHP and mPFC. **b** Immunohistochemical analyses of dHP (bottom) and mPFC (top) sections showing the expression of mCherry in dHP CA1 neurons and dHP-recipient mPFC neurons after transneuronal transfer of Cre recombinase. Sections were costained for mCherry (red) and the neuronal marker NeuN (green). Scale bars, 1 mm. **c**–**f** Open-field activity of mice was measured 30 min after CNO injection. **d** Sample path recordings of mice during the first 5 min (left) and entire 30-min period (right) in the OFT. Quantification of the distance moved across 5-min time bins (**c**), the entire 30-min period (**e**), and thigmotaxis (**f**) in the open field box. Suppression of dHP CA1 neurons increased the time spent immobile in the TST (**g**) and FST (**h**). Chemogenetic activation of dHP-recipient mPFC neurons blocked the effect of dHP CA1 suppression on TST (**g**) and FST (**h**) behaviors. The TST and FST were conducted 1 h and 30 min, respectively, after CNO injections, and the interval between the TST and FST was 2 weeks. (**e**−**h**) *p < 0.05, **p < 0.01, ***p < 0.001, n.s., not significant (*p* ≥ 0.05).

We hypothesized that if neuronal activity in the dHP affects the coping strategy to inescapable stress through projections to the mPFC, manipulation of the activity of the mPFC would abolish the effects of altered neuronal activity in the dHP on stress-coping behaviors. To test this hypothesis, we first confirmed the Cre recombinase activity of WGA-Cre in infected cells. The protein expression of EGFP was detected in the dHP CA1 neurons of the mice infected with a mixture of AAV-hSyn1-mCherry-IRES-WGA-Cre and AAV-hSyn-DiO-EGFP in the dHP (Supplementary Fig. 10). We then coexpressed hM4Di and WGA-Cre in dHP CA1 neurons, and hM3Dq-mCherry or mCherry was expressed in dHP-recipient mPFC neurons via transneuronal transfer of WGA-Cre (Fig. 7a, b and Supplementary Fig. 11). Manipulation of the chemogenetic activity of these neurons did not significantly affect the behavior of the mice in the OFT (Fig. 7c–f). However, consistent with the behavioral effects of muscimol infusion into the dHP CA1 region (Fig. 3l), chemogenetic activity suppression of dHP CA1 neurons increased immobility time during the TST and FST (Fig. 7g, h). Intriguingly, chemogenetic activation of dHP-recipient mPFC neurons abolished the behavioral effects induced by activity suppression of dHP CA1 neurons and induced active coping behaviors in the TST and FST. These results suggest that neural activity in the dHP affects stress-coping behaviors via excitatory projections to the mPFC.

## Discussion

The present study suggested that neural activity in the dHP affects coping strategies during inescapable stress by modulating mPFC activity in mice. Manipulations of inhibitory synaptic transmission and the activity of interneurons in the dHP CA1 area bidirectionally modified stress-coping behaviors without affecting general locomotor activity or anxiety. The dHP projects monosynaptic excitatory exons to the mPFC, and stimulation of the dHP increases neuronal activity in the mPFC. Chemogenetic activation of mPFC neurons in the dHP-mPFC circuit abolished the behavioral effects of activity suppression in the dHP. Thus, in addition to cognitive processing, the dHP seems to affect coping strategies for stress rather than anxiety or anhedonia through the dHP-mPFC circuit. Ketamine rapidly increased the neural activity of CA1 principal cells in both the dHP and vHP. These observations further indicate that the increased activity of CA1 pyramidal neurons in the dHP may contribute to the antidepressant effects of ketamine.

Immobile behavior in the TST and FST is believed to represent passive coping behavior to acute inescapable stress or behavioral despair by reflecting a failure to persist in escape-directed behavior^36–38^. Behavioral changes in these tests may result from behavioral adaptation or habituation, at least during the FST^39^. In the TSTs and FSTs, which were conducted in different testing rooms, the manipulation of the inhibitory circuitry or activity suppression of neurons in the dHP produced similar effects on immobile behaviors. In addition, no significant difference in immobility time was observed between the first and second TSTs of the saline-treated mice (Fig. 1a), indicating that TST behaviors are unlikely to be affected by learning or habituation.

Defects in the GABAergic circuitry and an increased synaptic excitation-inhibition (E/I) ratio in the brain were found to be associated with depressive disorders^40,41^. Decreased GABA and increased glutamate levels have been observed in the occipital cortex of patients with major depressive disorder (MDD)^42^, and a postmortem study revealed fewer GABAergic neurons in the dorsolateral PFC of patients with MDD^43^. Other studies have revealed decreased levels of glutamatergic metabolites (glutamate and glutamine) in the mPFC, anterior cingulate cortex (ACC), and/or hippocampus of patients with MDD compared with healthy controls^44–48^. These findings suggest that the E/I ratio in individuals with depression differs depending on the brain area. A preclinical study showed that heterozygous deletion of the GABA_A_R γ2 subunit (γ2^+/−^) induces depression-like behaviors in mice^33^. Interestingly, γ2^+/−^ mice exhibit a homeostatic-like reduction in the expression levels of glutamate receptors, as well as functional impairment of glutamatergic synapses in the hippocampus and mPFC. These glutamatergic defects and depression-like behaviors in the γ2^+/−^ mice were reversed by ketamine^33^. In addition to GABAergic defects, glutamatergic defects, such as reduced spine density in pyramidal neurons and impaired excitatory synaptic transmission and plasticity in the hippocampus, have been consistently observed in stressed rodents^9,49–53^. These alterations in excitatory and inhibitory synapses may result in abnormal neuronal activity in the hippocampus and contribute to depression-like behavior in stressed rodents.

Our results show that altered synaptic inhibition or neuronal activity in the dHP is sufficient to modulate immobile/despair-like behavior in the TST and FST. Consistent with our results, a recent clinical study reported that an increase in hippocampal activity in patients with major depressive disorder who received repetitive transcranial magnetic stimulation treatment was specifically associated with the amelioration of depressive symptoms but not anxiety or sleep quality^54^. In addition, tamoxifen-induced removal of GluN2A in the adult mouse brain or GluN2A knockdown in the dHP reduced despair-like behavior without affecting locomotor activity or anxiety^55^. Although whether the dHP-mPFC circuit is associated with the antidepressant-like behavioral effects of GluN2A knockdown is unknown, these observations suggest that diverse symptoms in depression, such as low mood, anxiety, anhedonia, and despair, likely arise from different brain areas and distinct neurochemical signaling.

As the direct activation of CA1 pyramidal neurons may induce epileptic activity in the dHP, we monitored behavioral responses to the decreased activity of dHP CA1 pyramidal neurons. In contrast to the suppression of inhibitory neuronal activity, chemogenetic suppression of dHP CA1 neurons, including pyramidal and inhibitory neurons, induced passive coping behaviors in mice. However, the activation of dHP-recipient mPFC neurons blocked the behavioral effects of dHP CA1 suppression. Notably, the activation of these mPFC neurons induced active coping behaviors despite decreased neural activity in the dHP, suggesting that the dHP affects coping behaviors through the dHP-mPFC circuit. Intriguingly, a recent study showed that inhibition of Drd1-expressing cells in the mPFC blocked the ketamine response, and optogenetic stimulation of these cells produced ketamine-like behavioral responses^56^.

Our study indicated that monosynaptic transmission from the dHP CA1 region to the mPFC is glutamatergic. Although dHP CA1 axons project to both excitatory and inhibitory neurons in the mPFC, optogenetic stimulation of exons from the dHP CA1 region induces spike firing in mPFC pyramidal neurons. Consistent with these results, stimulation of dHP CA1 neurons in vivo resulted in c-Fos induction and increased calcium transients in mPFC pyramidal neurons. Given that increased PFC activity is followed by the onset of struggling and is maintained during periods of struggling in the TST^57^, our results indicate that increased neuronal activity in the dHP may induce active avoidance behavior by increasing neural activity in the mPFC. Inactivation of the vHP or inhibition of TrkB phosphorylation in the vHP blocks the sustained but not acute antidepressant effects of ketamine^17^. Consistent with this observation, we found that ketamine administration caused rapid c-Fos induction in the dHP and vHP CA1 pyramidal populations regardless of TrkB receptor inhibition. Collectively, these findings suggest that the rapid antidepressant effects of ketamine can be attributed, either in full or in part, to the increased activity of pyramidal neurons. The selective suppression of the sustained antidepressant effect of ketamine by the TrkB receptor antagonist ANA-12 further supports this finding. Ketamine has been suggested to induce long-lasting (>24 h) increases in excitatory synaptic transmission in CA1 pyramidal neurons^58^. This finding indicates that an increased E/I ratio^58^ or increased signal-to-noise ratio caused by BDNF-induced neurotropic and neuroplastic changes^59,60^ may contribute to the long-lasting antidepressant effects of ketamine.

Interestingly, infection of the dHP CA1 region with the shGabrg2 virus induced molecular changes similar to those induced by ketamine administration, except for mTOR phosphorylation. In addition, these molecular changes were not observed in the vHP. Previous reports have shown that there are intrahippocampal projections from the dorsal CA2 to the ventral CA1 and from mossy cells in the vHP to dorsal dentate granule cells^61–63^. Although the projections from the dorsal CA1 to the vHP subfields are unclear, we did not observe EGFP signals in the vHP of the mice expressing shGabrg2 in the dHP CA1 (Supplementary Fig. 4). Synaptic and molecular changes in the dHP induced by GABA_A_R γ2 knockdown indicate that disinhibition or an increase in the E/I ratio is sufficient to induce eEF2 dephosphorylation and BDNF elevation in the absence of ketamine and that additional mechanisms contribute to ketamine-induced mTOR phosphorylation. As ketamine directly binds to TrkB receptors and facilitates synaptic localization and activation of TrkB receptors via BDNF^15^, direct activation of TrkB receptors with synaptic disinhibition may underlie ketamine-induced activation of the phosphatidylinositol 3‑kinase (PI3K)/protein kinase B (Akt) signaling pathway and mTOR phosphorylation^9^.

Further investigations are needed to identify ketamine-induced modifications in dHP and dHP inputs to mPFC neurons.

## Supplementary information

Supplementary Information

Supplementary Table 1
