# Supplementary material for: Activity in the dorsal hippocampus-mPFC circuit modulates stress-coping strategies during inescapable stress
Source: Exp Mol Med. 2024 Sep 2;56(9):1921–35. doi: 10.1038/s12276-024-01294-z (PMC11447212; doi:10.1038/s12276-024-01294-z)
Supplement: Supplementary file 1 — Supplementary Information [file 12276_2024_1294_MOESM1_ESM.pdf]

Supplementary Information

Activity in the dorsal hippocampus-mPFC circuit modulates stress-coping strategies during inescapable stress

Sang Ho Yoon, Woo Seok Song, Geehoon Chung, Sang Jeong Kim, and Myoung-Hwan Kim

Supplementary figures (Fig. 1-12)

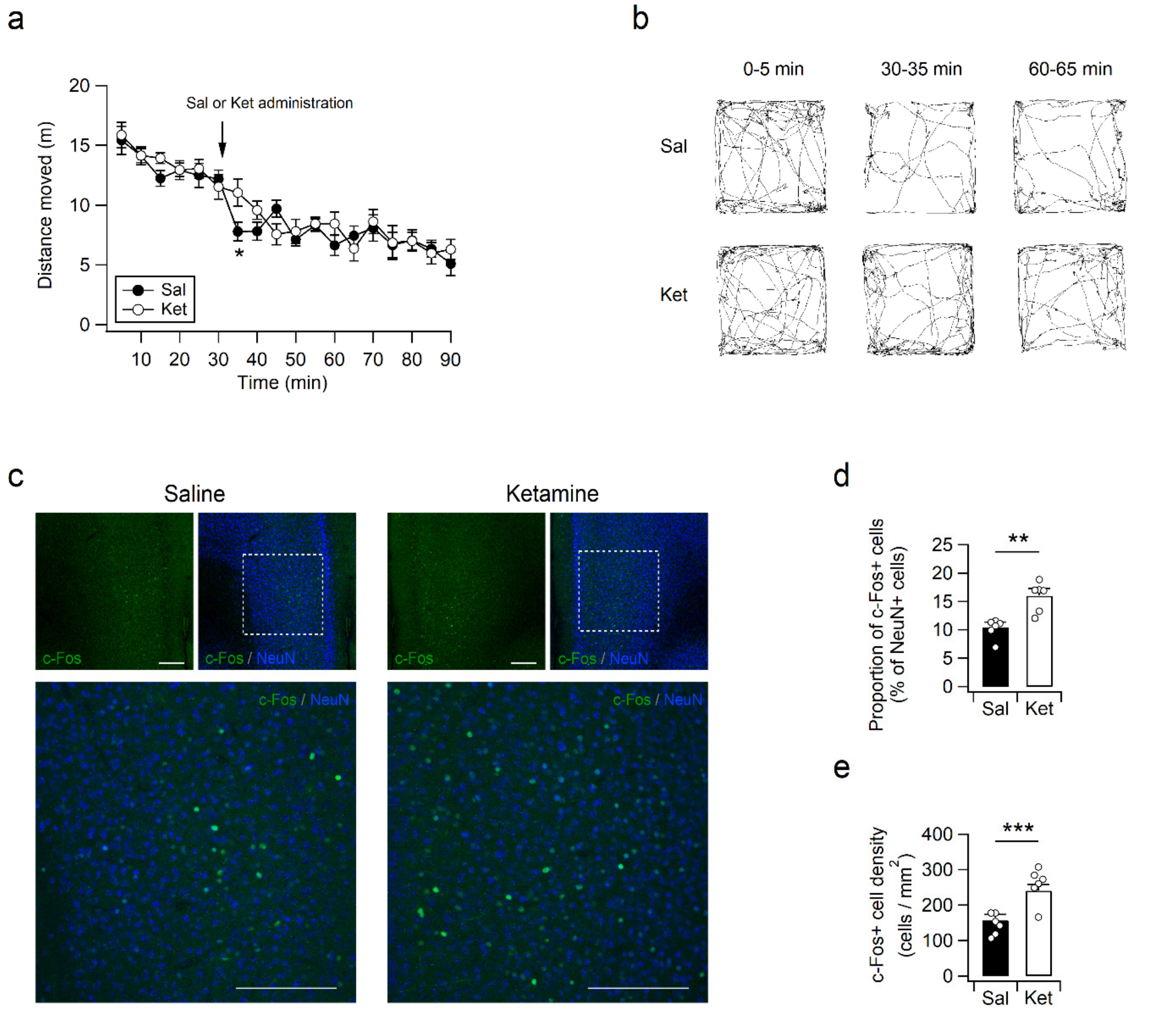

Supplementary Fig. 1: Enhanced c-Fos expression in the mPFC of ketamine-treated mice. (a) Open field activities of ketamine- or saline-treated mice. Mice received ketamine (5 mg/kg) or saline 30 min after baseline activity recording. (b) Sample path recordings during the first 5 min, and immediately after (30-35 min) and 30 min after (60-65 min) drug administration. (c) Ketamine induced rapid enhancement in c-Fos (green) expression in the mPFC. NeuN (blue) was used to identify neurons in the mPFC. The bottom shows magnified images indicated by the dotted white boxes in the upper panels. Scale bars, 200 μm. (d) The number of cells expressing c-Fos was normalized to the total number of NeuN+ cells in the mPFC. (e) Bar graphs represent the density of cells expressing c-Fos in the mPFC of mice receiving saline or ketamine. N = 6 slices from 3 mice.

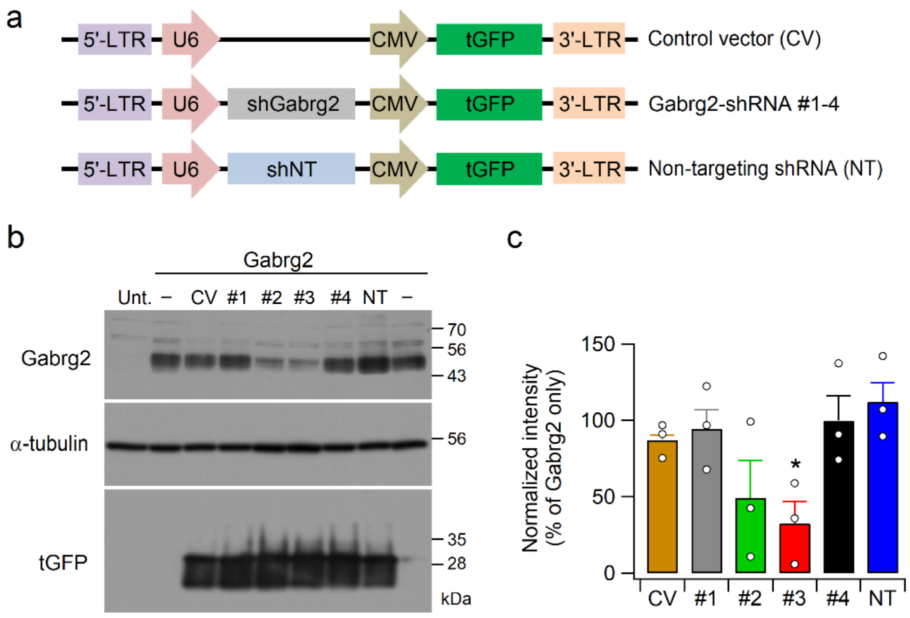

Supplementary Fig. 2: Western blot analysis of Gabrg2 knockdown efficiencies of four different shRNA sequences (sh#1-4) in HEK293T cells. (a) Schematic representation of the shRNA constructs. (b) Representative western blots for Gabrg2, α-tubulin, and TurboGFP (tGFP) obtained from HEK293T cell lysates at 72 h post-transfection. The control vector (CV) expresses tGFP without a hairpin sequence. The non-targeting (NT) shRNA contains a tGFP expression cassette and the hairpin with surrounding sequences that do not overlap with any known mouse gene (see Materials and Methods for details). α-tubulin and tGFP were used as loading and transfection controls, respectively. Unt., untransfected control HEK293T cells. (c) Quantification of the Gabrg2 knockdown efficiency in transfected cell lines.

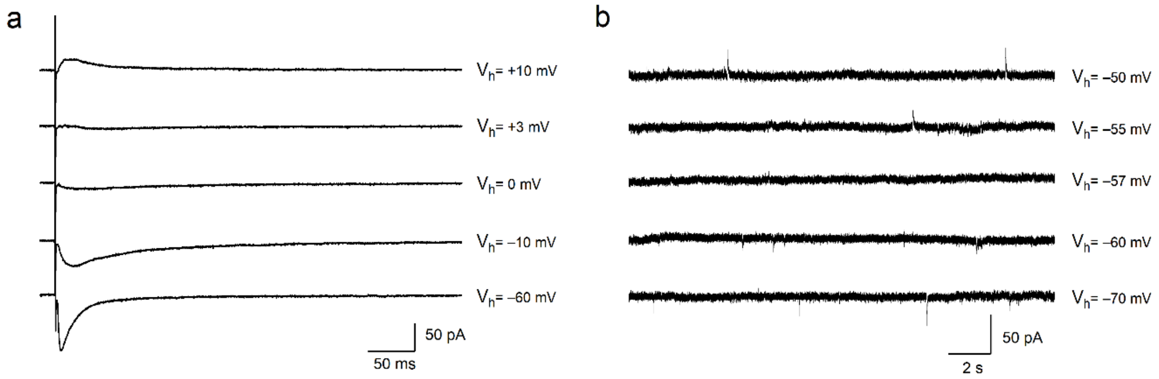

Supplementary Fig. 3: Determination of the reversal potentials for EPSCs and IPSCs in CA1 pyramidal neurons. (a) Sample traces of evoked EPSCs in the presence of the $GABA_AR$ blocker picrotoxin in the bathing solution. The holding potentials (V_h) are shown with each trace. Recording at +3 mV leads to negligible evoked EPSCs owing to the absence of a driving force for cations. (b) Example traces of spontaneous IPSCs recorded from CA1 pyramidal neurons in the presence of the AMPAR blocker NBQX and NMDAR blocker AP-5. The reversal potential of spontaneous IPSCs was −57 mV.

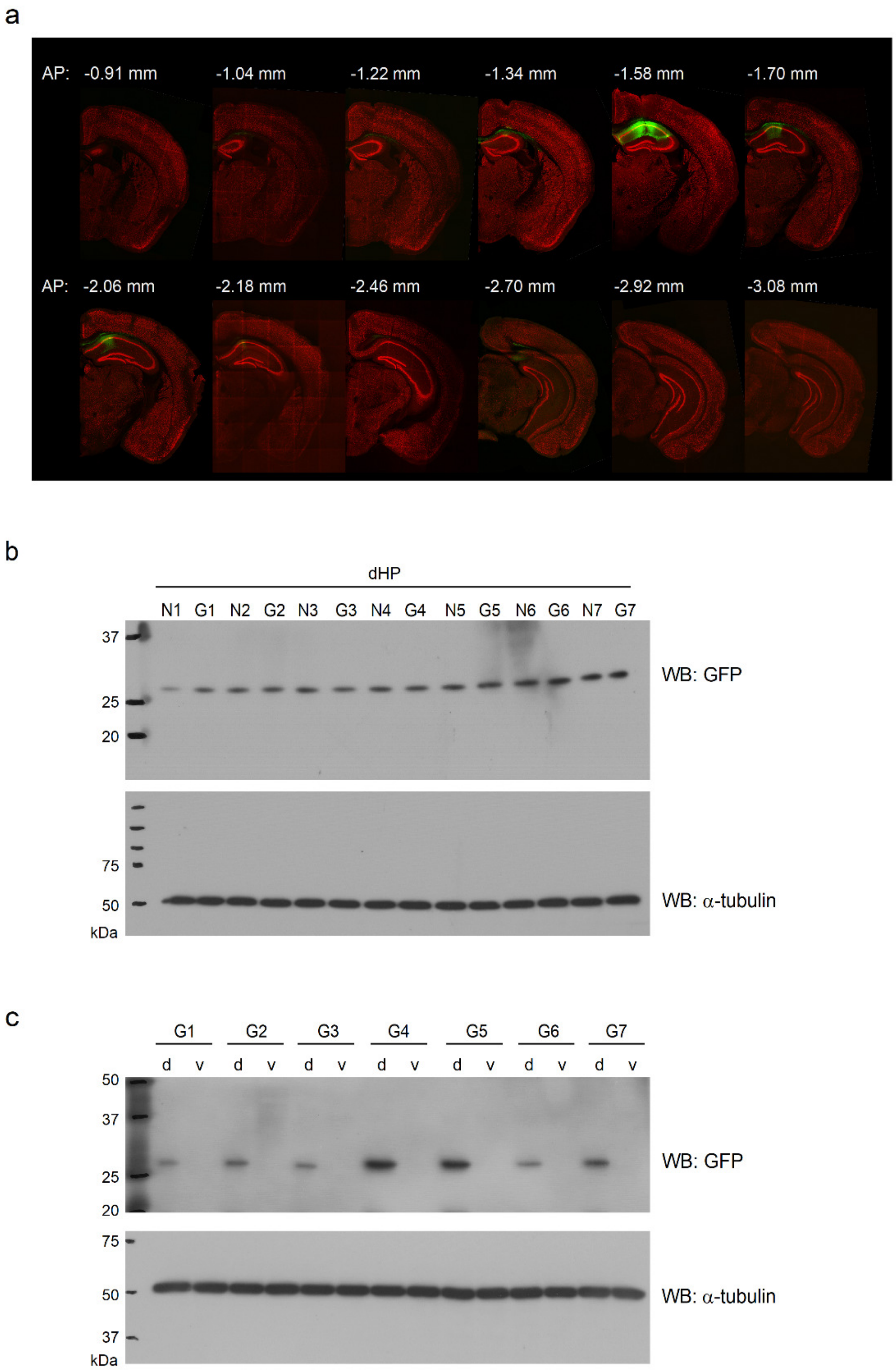

Supplementary Fig. 4: Histochemical and western blot analyses of EGFP distribution show selective infection of lentivirus in the dHP. (a) Series of immunofluorescent images of coronal sections co-stained with EGFP (green) and NeuN (red) show the distribution of lentivirus-infected cells (green) in the mouse brain. Numbers indicate anterior/posterior (AP) coordinates relative to the bregma. (b) The expression of GFP was detected in the dHP of mice infected with virus expressing non-targeting shRNA (N) or shGabrg2 (G). (c) Western blot images showing the selective expression of GFP in the dHP but not vHP of mice infected with lentivirus expressing shGabrg2. D and V represent dHP and vHP, respectively.

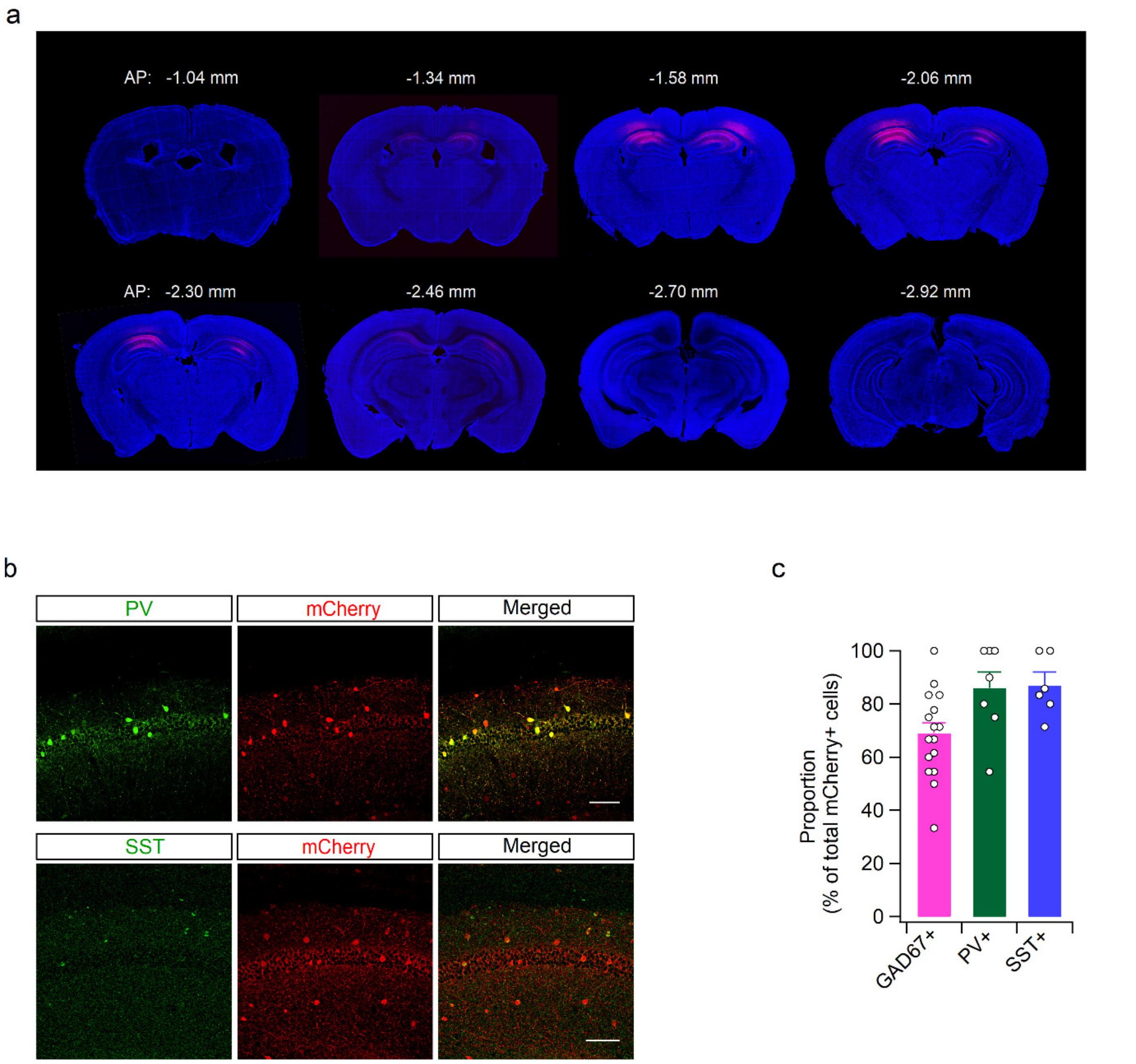

Supplementary Fig. 5: Expression of mCherry in the PV- and SST-expressing neurons in the dHP CA1 area of Vgat-Cre mouse. (a) Series of mCherry immunofluorescent images of coronal brain sections show the distribution of cells expressing mCherry (red) in the Vgat-Cre mouse. mCherry signals are mainly located in the CA1 subfields of the dHP. DAPI (blue) was used for the identification of brain regions. Numbers indicate anterior/posterior (AP) coordinates to Bregma. (b) Hippocampal sections from Vgat-Cre mice infected with DiO-DREADDs-mCherry were co-immunostained with PV/mCherry (top) or SST/mCherry (bottom) antibodies. Scale bars, 100 µm. (c) Quantification of mCherry expressing cells in the hippocampal CA1 regions. mCherry signals were detected in 69%, 86%, and 87% of GAD67-, PV-, and SST-positive cells, respectively. N = 16 slices from 5 mice (GAD67), 7 slices from 3 mice (PV), and 6 slices from 3 mice (SST).

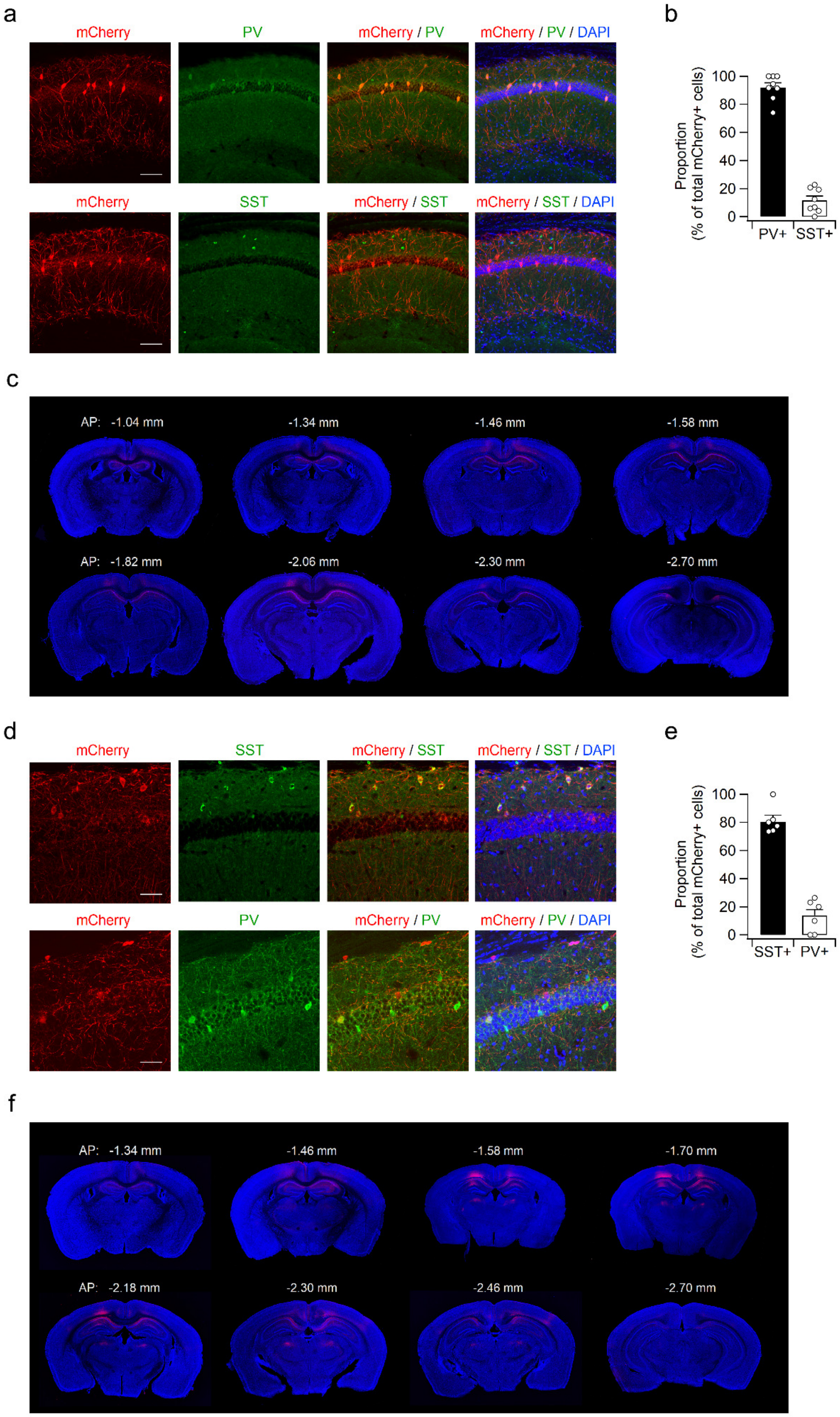
a
mCherry
PV
mCherry / PV
mCherry / PV / DAPI
mCherry
SST
mCherry / SST
mCherry / SST / DAPI
b
Proportion
(% of total mCherry+ cells)
100
80
60
40
20
0
PV+
SST+
c
AP: -1.04 mm
-1.34 mm
-1.46 mm
-1.58 mm
AP: -1.82 mm
-2.06 mm
-2.30 mm
-2.70 mm
d
mCherry
SST
mCherry / SST
mCherry / SST / DAPI
mCherry
PV
mCherry / PV
mCherry / PV / DAPI
e
Proportion
(% of total mCherry+ cells)
100
80
60
40
20
0
SST+
PV+
f
AP: -1.34 mm
-1.46 mm
-1.58 mm
-1.70 mm
AP: -2.18 mm
-2.30 mm
-2.46 mm
-2.70 mm

Supplementary Fig. 6: Preferential expression of DREADDs-mCherry in the PV- or SST-positive cells in the dHP. (a) Co-immunostainings of hippocampal sections with PV/mCherry (top) and SST/mCherry (bottom) show preferential expression of mCherry in the PV-expressing cells. Scale bars, 100 µm. (b) PV- or SST-positive signals were detected in 92% and 12% of mCherry-expressing cells in the dHP CA1 area of a PV-Cre mouse. N = 8 slices from 4 mice. (c) Series of mCherry immunofluorescent images of coronal brain sections from a PV-Cre mouse showing the distribution of mCherry (red) in the dHP CA1 area. AP indicates anterior/posterior to the bregma. (d) Co-immunostainings of hippocampal sections with SST/mCherry (top) and PV/mCherry (bottom) revealing predominant expression of mCherry in SST-expressing cells. Scale bars, 50 µm. (e) Quantification of mCherry-expressing cells shows the predominant expression of mCherry in SST-positive cells (81%) compared with PV-positive cells (13%) in the dHP CA1 area. N = 6 slices from 3 mice for each cell type. (f) The distribution of mCherry-expressing cells in a mouse brain infected with a mixture of AAV-DiO-DREADDs-mCherry and AAV-SST-Cre was determined by immunohistochemical staining with mCherry antibodies. DAPI (blue) was used to identify brain regions and structures (a, c, d, f).

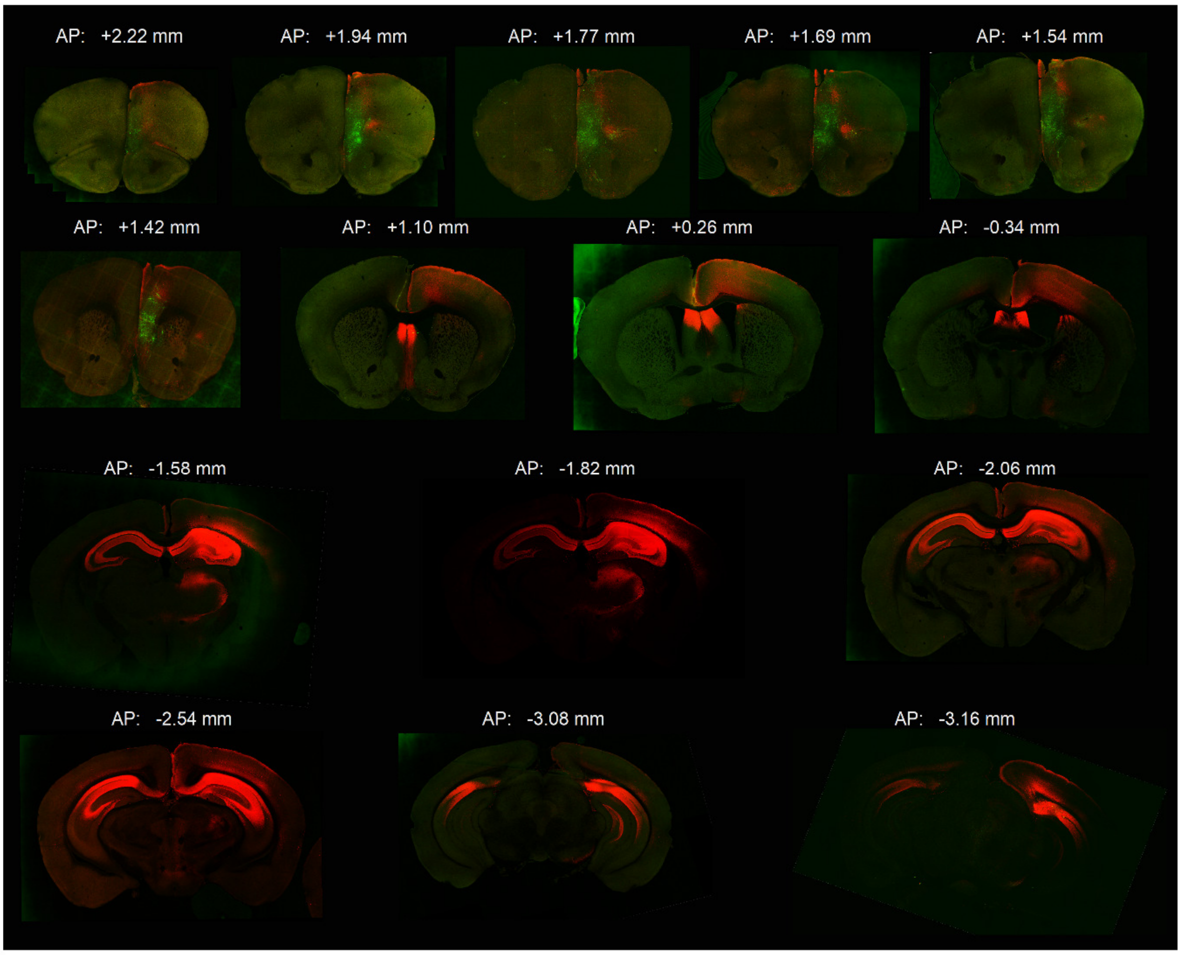

Supplementary Fig. 7: Transneuronal labeling of the dHP-mPFC circuit. AAV DJ-hSyn1-mCherry-IRES-WGA-Cre and AAV-hSyn1-DiO-EGFP were unilaterally injected into the dHP CA1 subfield and mPFC, respectively. Co-immunostaining of brain sections with EGFP and mCherry antibodies shows EGFP-expressing cells in the mPFC. mCherry signals were detected in the somata of dHP CA1 neurons and their fibers that innervate the contralateral CA1 area, lateral septal nucleus, anterior cingulate cortex, and mPFC.

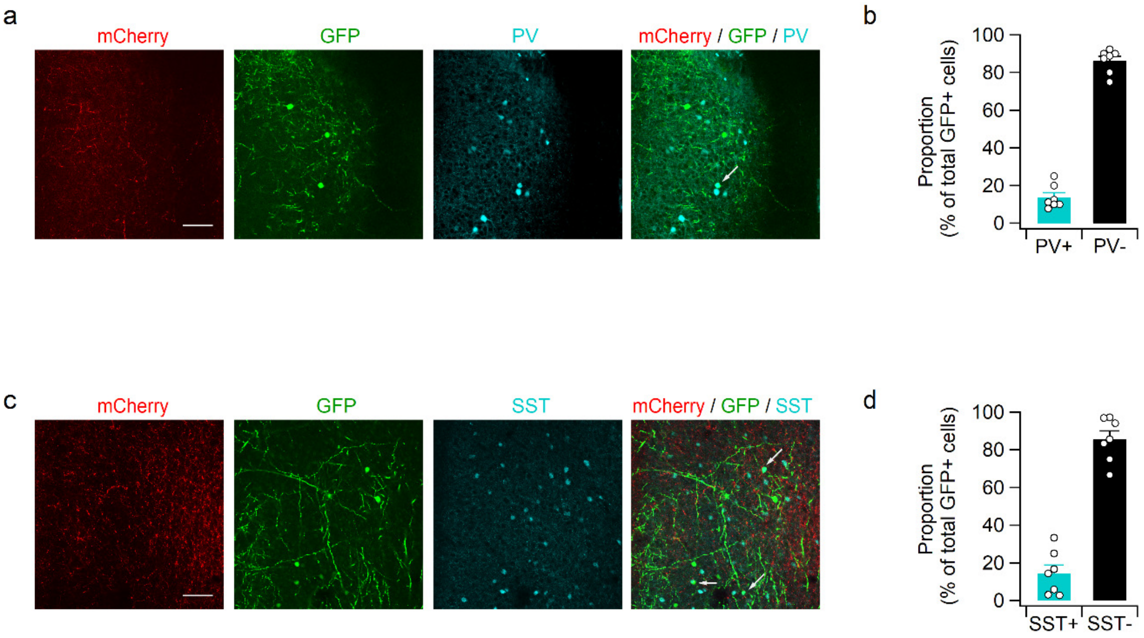

Supplementary Fig. 8: CA1 neurons in the dHP project to excitatory and inhibitory neurons in the mPFC. (a-d) Unilateral injections of AAV DJ-hSyn1-mCherry-IRES-WGA-Cre and AAV-hSyn1-DiO-EGFP into the dHP CA1 and mPFC, respectively, resulted in the expression of EGFP in the mPFC cells including PV- and SST-expressing cells. Arrows indicate GFP-positive cells expressing PV or SST. Scale bars, 100 µm (a, c).

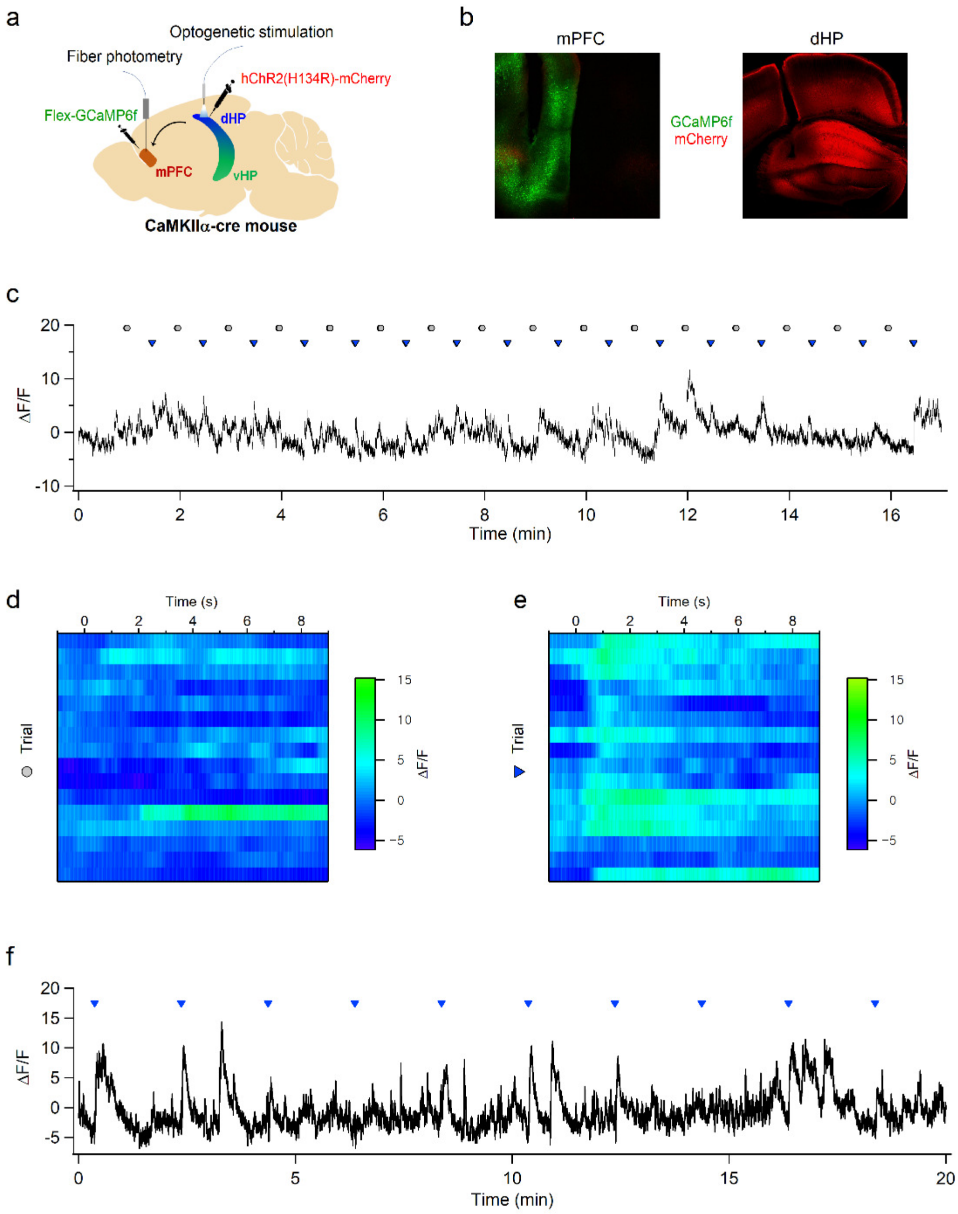

Supplementary Fig. 9: Optogenetic stimulation of dHP CA1 neurons induces calcium transients in excitatory neurons in the mPFC. (a) Experimental design for simultaneous fiber photometry recording and optogenetic stimulation. (b) Immunofluorescent images of coronal brain sections showing the cannula placement and the expression of GCaMP6f and mCherry in the mPFC and dHP CA1, respectively. (c) Representative fluorescence ΔF/F trace recorded in the mPFC from a freely moving CaMKII-Cre mouse. Light stimulation (5 mW, 20 Hz, 1 s) was delivered to the dHP every 1 min and is indicated by blue triangles. Gray circles indicate the midpoint between each stimulation. (d, e) GCaMP6f traces during the midpoint between each stimulation (d; gray circles in panel c) and the peri-stimulation (e; blue triangles in panel c) are represented by trial-by-trial heatmaps. The vertical axis represents trials, and the color scale indicates ΔF/F. (e) Stimulation was delivered at 0 s. (f) A sample fluorescence ΔF/F trace recorded in the mPFC of GCaMP6f-expressing mouse in response to high frequency optogenetic stimulation (5 mW, 100 Hz, 20 stimulations) of dHP CA1 neurons. Light stimulation is indicated by blue triangles.

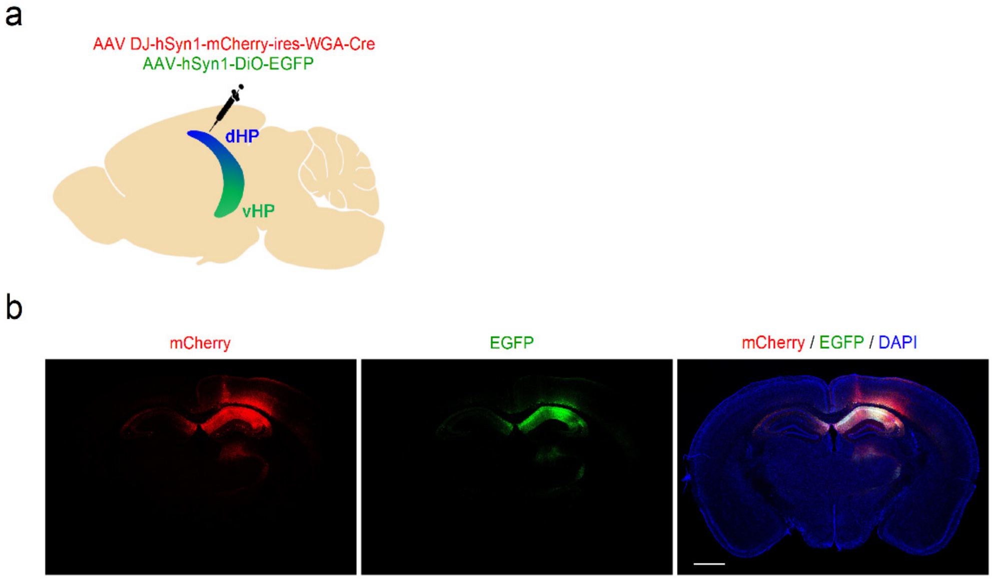

Supplementary Fig. 10: Cre recombinase activity was detected in dHP neurons infected with AAV-expressing WGA-Cre. (a) A mixture of AAV-hSyn1-mCherry-IRES-WGA-Cre and AAV-hSyn-DiO-EGFP was injected unilaterally into the dHP. (b) Immunofluorescent images of coronal brain sections showing the expression of EGFP in the dHP via WGA-Cre. DAPI (blue) was used to identify brain regions and structures. Scale bar, 1 mm.

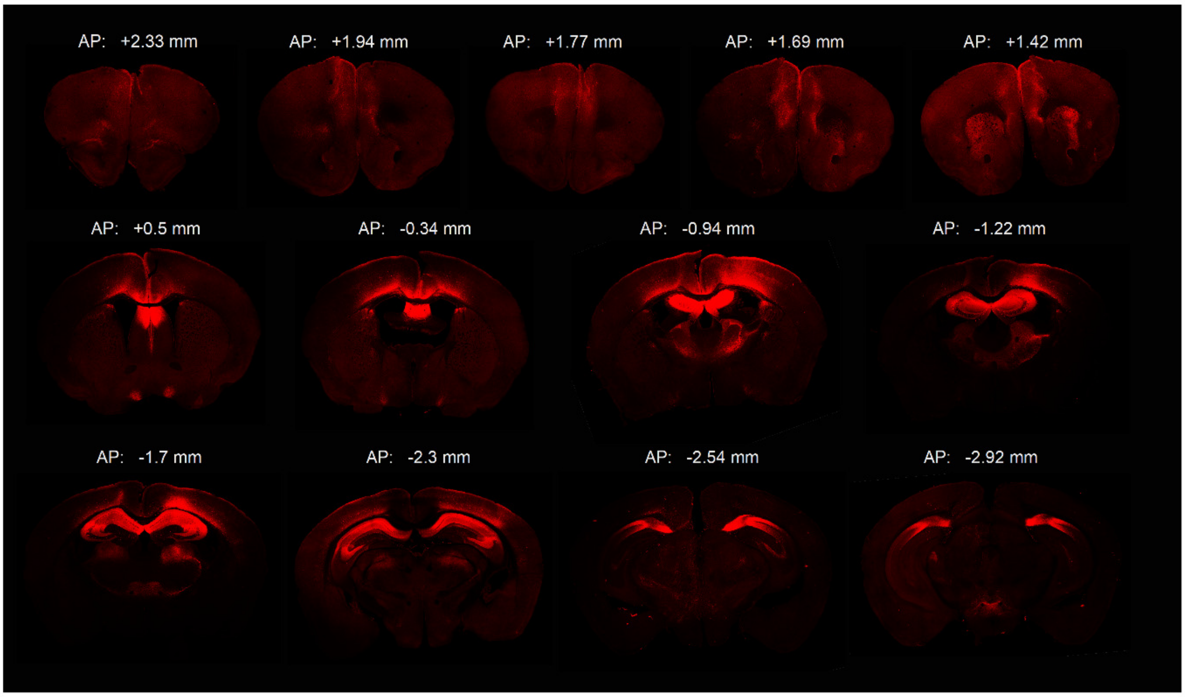

Supplementary Fig. 11: Histochemical analysis of mCherry distribution in the mouse brain. AAV-DiO-hM3Dq-mCherry and a mixture of AAV DJ-hSyn1-mCherry-IRES-WGA-Cre and AAV-DiO-hM4Di-mCherry were bilaterally injected into the mPFC and dHP CA1 subfield, respectively. Series of immunofluorescent images of coronal brain sections showing the expression of hM3Dq-mCherry in dHP-recipient mPFC neurons via transneuronally transferred WGA-Cre.

Fig. 1b

S: saline, K: ketamine

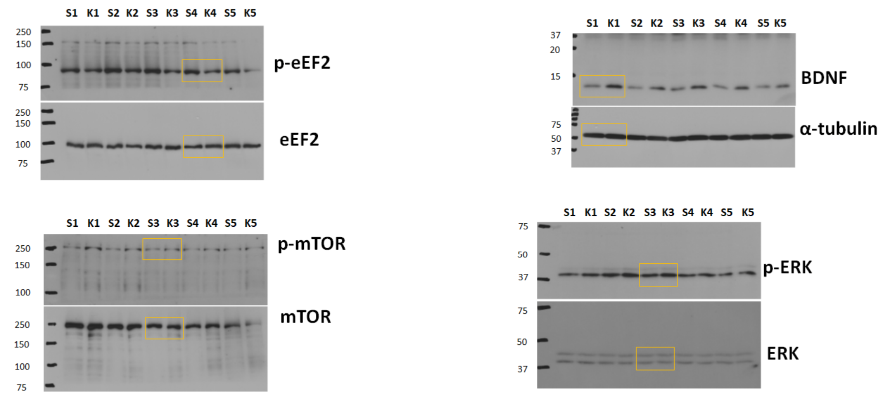

Fig. 3b (dHP)

N: shNT, G: shGabrg2

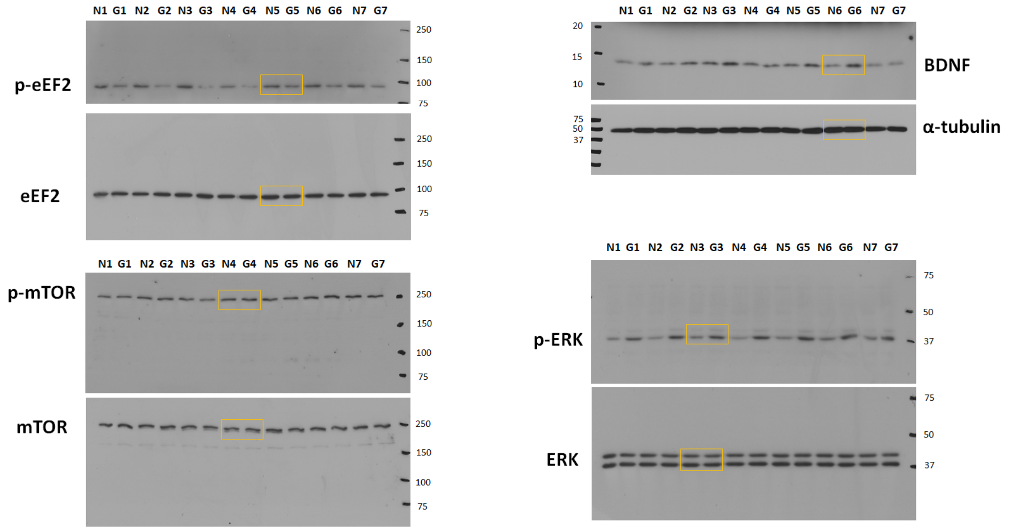

Fig. 3b (vHP)

N: shNT, G: shGabrg2

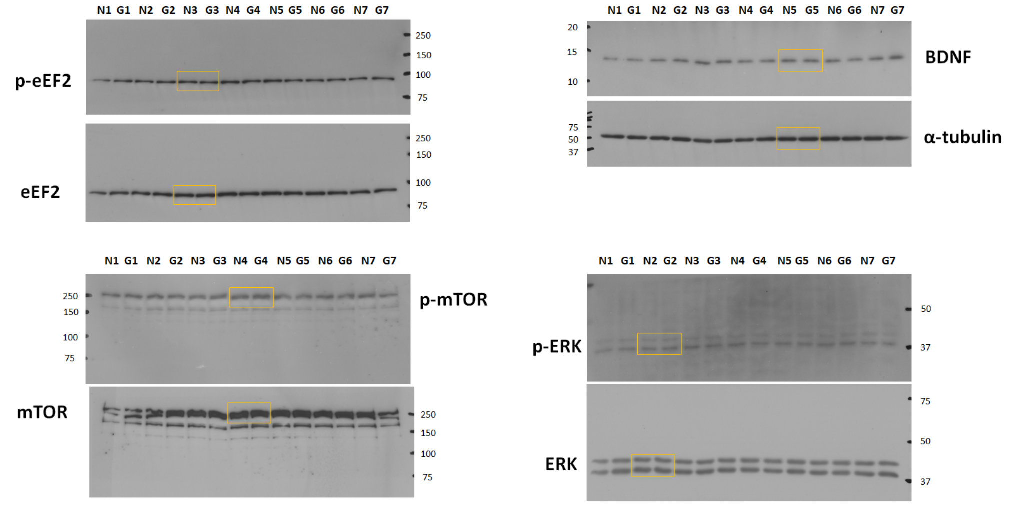

Supplementary Fig. 12: Uncropped western blot images presented in the main figures.
